# Supplementary material for: Exploring the feasibility of integrating health, nutrition and stimulation interventions for children under three years in Nepal’s health system: A qualitative study
Source: PLOS Glob Public Health. 2023 Apr 28;3(4):e0001398. doi: 10.1371/journal.pgph.0001398 (PMC10146516; doi:10.1371/journal.pgph.0001398)
Supplement: S1 Table — (PDF) [file pgph.0001398.s001.pdf]

S1 Table: Sampling framework

Participants	Number of SSI	Number of FGD	Number of participants	Criteria of selection (By location)	Location of interview/ discussions
**A. Caregivers**					
Father of <3 years of age child	3		3	a) Rural Municipality	Dhanusha
	3		3	b) Urban Municipality	Dhanusha
Mother of <3 years of age child	3		2	a) Rural Municipality	Dhanusha
	4		5	b) Urban Municipality	Dhanusha
Grandmother of <3 years of age grandchild	2		2	a) Rural Municipality	Dhanusha
	3		2	b) Urban Municipality	Dhanusha
**B. Health service providers**					
Health workers - health coordinators or health assistant or auxiliary nurse midwife	2		2	a) Rural Municipality	Dhanusha
	2		2	b) Urban Municipality	Dhanusha
Female Community Health Volunteers (FCHVs)		1	7	a) Rural Municipality	Dhanusha
		1	7	b) Urban Municipality	Dhanusha
**C. District stakeholders**					
Nutrition/Education programme manager	1		1	Non-government stakeholder implementing Nutrition or Education programme	Dhanusha
Health Facility Operation and Management Committee (HFOMC)		1	6	a) Rural Municipality	Dhanusha
		1	6	b) Urban Municipality	Dhanusha
**D. National stakeholders**					
Nutrition specialist	2		2	Non-government stakeholder from UN organisation (nutrition and early childhood development sector), civil society, INGOs	Kathmandu (online via Zoom)
Early childhood development specialist	2		2		Kathmandu (online via Zoom)
**E. Policymakers**					
Province health representative	1		1	Provincial level government representative	Dhanusha
National governmental representative	2		2	National level government representatives from MoHP and MoEST	Kathmandu (face to face and online via Zoom)
**Total**	**30**	**4**	**55**		

Abbreviations: SSI Semi-structured Interview; FGD Focus Group Discussion; UN United Nations, INGO International non-governmental organisation, MOHP Ministry of Health and Population, MoEST Ministry of Education, Science and Technology
