# Supplementary material for: Exploring the feasibility of integrating health, nutrition and stimulation interventions for children under three years in Nepal’s health system: A qualitative study
Source: PLOS Glob Public Health. 2023 Apr 28;3(4):e0001398. doi: 10.1371/journal.pgph.0001398 (PMC10146516; doi:10.1371/journal.pgph.0001398)
Supplement: S2 Table — (PDF) [file pgph.0001398.s002.pdf]

S2 Table: Supporting qualitative data

Participant's responses
**Theme 1 The case for integrated intervention**
**Sub-theme 1.1 Many see nutrition and play are interrelated**
**Caregivers were interested in play programmes that supports the growth and development of their children.**<br><br>"We play with the child according to our choice but if INGOs (International Non-Governmental Organisations) will bring a programme related to the playing activities according to them then they might teach games in a technical way so that children can learn something. What I will teach will be a different thing and what professional will teach that will be a different thing. They will teach related things. They will teach with full knowledge and that is a good thing. That kind of programme must come." Father, SSI 3
**Several health workers, stakeholders and policymakers highlighted that nutrition and stimulation were interrelated.**<br><br>"Both nutrition and play programmes are like fingernails and muscles. It will be good to run both programmes combined. When children are given nutrition, they will just eat and sit. If they are only taught to play, they will not get enough nutrition. If they are taught to play, then when they sweat, they will need nutritious food to provide strength to their body. Children will get tired when they spend energy and become weak and need nutritious food." Health assistant, SSI 22
**Caregivers, health workers, stakeholders and policymakers said that playing enables children to learn quickly, improve their thinking capacity, and do better in school later.**<br><br>"Their brain will develop. Like when he shakes a rattle (*Jhunjhuna*) his capacity of hearing sound will increase. He will think like from where is the sound coming, and another thing is he tries to find the exact location from where the sound is coming, "Oh from that place the sound is coming" in this way slowly there will be development, which means their thinking capacity increases." Father, SSI 3<br><br>"P: Child will use brain and develop. They would not be dumb and lazy. I: So, would not be dumb and lazy, what else? P: They will have a brain and will study. They will become smart, clever and good. They will be able to keep calculations. Sister, these are things; they won't be dumb. If they do not play, they will be slow. I: But how much does he learn through games? P: A lot. The child will do as we do with him. I: What will the child do? P: Children will study and use their brains. They will do the governmental job after studying and will become clever. If children have a sharp brain, they will earn nicely, able to eat and survive. These are things." Mother, SSI 8
**Sub-theme 1.2 Informal play already occurs in the community, but is not always connected to learning**
**Health workers, stakeholders and a policymaker said that caregivers do not play with them thinking that play will help in their children's development.**<br><br>"In the village, parents are not aware. Very few parents think that children must be involved in games. There are very few parents who play with their children for their health. But unknowingly, they play

**Participant's responses**
with their child to distract them and to spend time. Even though they do not know the importance of playing, they play with their child. Brothers and sisters play with babies to distract them or to prevent them from crying, but they do not know the importance of play. Parents play with their child in the traditional way not because they understand its importance that games improve physical and mental development of the child." Health assistant, SSI 22
**Caregivers and Female Community Health Volunteers (FCHVs) said mothers play with their children when they have a free time, the children play mostly by themselves.**<br><br>"I: But how much time does a guardian give a child to play? For instance, play with toys or talk with them, teach them? P5: Anytime they get free. P6: When they have enough time they play and if they do not have free time they do not play with their child. P3: In remote (village), mother take their child to field where they work. There children play in field with sand and mud. P4: If no one is there to play with the child at house, mother takes child everywhere they go. I: Children play themselves in the field. P3: Yes, they do […] P7- Mother must make breakfast and if husband must leave for duty, they also must prepare lunch. She gives toys to the child and the child play themselves." FCHVs, FGD 2
**Sub-theme 1.3 Gaps in the implementation of the existing policy for integrated ECD interventions**
**National stakeholders and policymakers said that Early Childhood Development (ECD) strategy has been developed, which will guide the local governments to deliver ECD activities in the community through intersectoral approach to improve ECD.**<br><br>"One of the challenges is kind of piecing all the bits together you know who's doing what around ECD and particularly stimulation and integration of stimulation because you have the Ministry of Education, the Ministry of women, children's, senior citizens, Ministry of Health, there are various NGOs and INGOs, government organisations etc. All are doing bits and pieces, and I think it's really timely that National Planning Commission (NPC) has led this development of overall national strategy, because hopefully that will bring all those different stakeholders together and provide that umbrella guidance for what needs to be done in the country. I think that's the good starting point and it's a recognition … as I said at the beginning that the government's acknowledgement of this is an issue and this is an important component of the childhood development, and it is not just seen as just one sector's responsibility but being seen as multiple sectors responsibility because the various component of the ECD crosses many different sectors." National stakeholder, SSI 27<br><br>"What we have envision in recently developed ECD strategy is that there should be a committee at every local government. There should be one primary child development committee in every local government. In that committee, health, protection and education officers train and present overall content. There will be arrangement for a child development coordinator. When there is a discussion in that committee these things (about integrating stimulation with ongoing nutrition programmes) should be additionally discussed, and they should integrate these things. Like I talked about developing ECD plan at local level. When they make these plans, they can discuss and add ECD programme." National stakeholder, SSI 28<br><br>"Currently, the NPC has developed a good strategy because it has set up a minimum standard like what should be the minimum qualification of the ECD facilitators, what is their job, what kind of training they should have received, duration of their training, ECD strategy has stated the minimum standards. I do expect that gradually this will move towards the implementation." National stakeholder, SSI 29

Participant's responses
**Stakeholders said that there is a culture of working in silos at the ministry level, which prevents an intersectoral approach to ECD.**<br><br>"At governmental level, they work in a silo. I have said it before too. We also used to work in a silo … Gradually, we are connecting. The Ministry of Health works separately and the Ministry of Education work in a different area. Women, children and the elderly ministry work in separate areas, and NPC works in a different area as well. They have different coverage networks (*Chettradhikar*). They say not to cross each other's area of coverage network. Because of this, things are challenging. Even when we were making the ECD strategy, there were comments like it is related to women and children and why this was not given to the Ministry of women, children and the elderly. I think the ECD evaluation (report published in 2018) also answers this "Why". The current status of children and women also speaks. We have an environment to work in a silo and limited understanding at the sectoral level. Like I gave an example of Multi-sectoral Nutrition Plan (MSNP) about the limited understanding. They think that ECD is under Multi-sectoral Nutrition Plan. I have been told by the governmental officer and UN staff that ECD falls under the nutritional plan. They have a difference in understanding and there is an environment of working in a silo. Until we break this, it is difficult to work in a holistic integrated manner." National stakeholder, SSI 28<br><br>"We don't have coordination between us. NPC think that ECD is a part of the multi-sectoral nutrition plan. What we say is nutrition is one of the components of ECD. Stimulation, health, protection-related aspects are not included in nutrition. This is a problem here. We developed ECD national strategy paper with the same NPC. It could have been done in one instead of two separate programmes. We have spent money and time to build that ECD strategy. It took us 1-2 years and similarly 1-2 years was spent to prepare MSNP I: Why is there this gap? How did it arise? P: We don't have a vision form the top level. […] NPC have prepared MSNP as well as ECD strategies. But they have not yet looked what could be merged while planning these. It is a problem." National stakeholder, SSI 30<br><br>"We need one integrated programme for ECD. This is what we are looking for. A new ECD strategy paper has also conceived this idea that we need such programme. This is not only the federal government's responsibility. This should be implemented by all three levels of government: federal, provincial and local. Within these governments, for example, in Kathmandu city, there are six types of ministries that are directly related but what they do is that they work separately. The ministry of education only sees the education sector ... ministry of women and children and elderly citizens is looking at protection only … there is no linkage between the ministries. But now the strategy paper is raising these things." National stakeholder, SSI 30
**A few national stakeholders and a policymaker said that some level of integration of stimulation component into national nutrition programme is in place.**<br><br>"There is already some integration […] You have the infant and young child feeding programme which also includes the use of multiple micronutrients powders etc. But part of that programme and part of the training of health workers of FCHVs on infant and young child feeding does include early stimulation. So that is reflected already in things like the information, education communication tools like flip chart, training manual training package where early stimulation is the part of those materials and of course as I said in the training of health workers and FCHVs there is a session on the importance and the role of early stimulation in positive nutrition and health outcomes and child development outcomes." National stakeholder, SSI 27

Participant's responses
"I: According to you, how can this stimulation programme combined with nutritional programme can be launched in the community? P: …We have stimulation as a part of an intervention within Maternal, Infant and Young Child Nutrition (MIYCN) training package. We talk and promote about active feeding babies by mothers. I: Is this still going on? Active feeding promotion? P: Yes. We have active feeding in our training package and have been training it to all the health workers and FCHVs as well. FCHV's are discussing it in group discussion with the mother's group as well, where female health volunteers give training about active feeding as a major component. We also provide awareness related to children's milestone which occurs respective to their age. It is also integrated in the training package. I: What are the things that have been included in the milestone? P: In milestone, we have included … when does the child start of grasping? … when they start to walk… before that when they have head movements or hold their head straight. These are the physical milestones under developmental milestones." Policymaker, SSI 25
**Sub-theme 1.4 Existing health and nutrition programmes provide opportunities for integration**
**Most health service providers, stakeholders and policymakers said that stimulation should be added in multiple ongoing health and nutrition programme.**<br><br>"I: In which nutrition programme should training programme be added to make it effective? P5: If we do not add in the currently ongoing programme then people will not come. So, we should conduct it together with our national vaccination programme then we could be including our targeted group and those children who have already received vaccination, they can be targeted through the Baal vita (micronutrient supplementation) distribution programme. We can also conduct stimulation programme along with this. Likewise, we have a Vitamin A distribution programme for children below the age of five which also includes our targeted age group that is children below three years. Similarly, we can conduct teaching programme about stimulation with these campaigns." HFOMC members, FGD 4<br><br>"In my personal view, we need to add stimulation programme in everything. We can see this clearly in Infant and young child feeding (IYCF) as well and it is there in IYCF that children should be given stimulation, but we concluded that it was not highlighted enough. […] In community, when they are distributing the therapeutic food or when supplying medicines for worms (anti-parasitic medicine) or multivitamins all they simply need to do is make them aware about basic things […] I see opportunities more than challenges." National stakeholder, SSI 28
**A few stakeholders said that the review of MSNP II provides a great opportunity for integrating stimulation into national nutrition programme.**<br><br>"MSNP is the national guiding document for nutrition programming in the country. So, if something is not in the MSNP, it won't get any priority. So, I think that's the first thing to be aware of. MSNP II is going to sort of come to the end of its so-called life at the end of next year and there will be already read in discussions about MSNP III and to get to MSNP III we need to do a thorough review of MSNP II, what's being implemented, how that contributed to any progress in nutritional status outcomes, childhood development etc. And I think this is the perfect opportunity to really make sure that ECD, early stimulation is incorporated in a next iteration of MSNP III is a great opportunity […] And the opportunity is also there because the multi-sectoral plan as its name is multi sectoral, it will help to define role of different sectors in how they can be supportive and accountable for these integrated approaches as well." National stakeholder, SSI 27

**Participant's responses**
**Theme 2: Integrated interventions in practice**
**Sub-theme 2.1 Intervention orientations and advertisements to promote participation**
**Caregivers, health service providers, and HFOMC members awareness of the significance of integrated interventions should be raised through orientations and advertisements at community gatherings.**<br><br>"In the ward, there should be a meeting, and then information about the programme should be discussed and then only all the children will be involved. There should be a meeting in every tole [area] … advertisement should also be done, and only it is possible to encourage participation." ANM, SSI 19
**Some health workers and HFOMC members said that the messages about integrated ECD intervention could be delivery through schools:**<br><br>"I: You have mentioned that school and teacher also can take lead. How can they participate in this?<br>P5: In school there are teachers. School teacher can teach children in school or students about: when children below three years are given nutritious food and are played with that would eventually help in growth and development of their brain and body. Now that child will go home and share this information with his father, mother and guardians. This would result in creating a positive impact on everyone. This can be a great medium of sharing information." HFOMC members, FGD 3<br><br>"I think the school has health education curriculum. Those teachers and focal person who are in school should be made aware of this programme about playing and learning and nutrition and orient them about the benefits of this programme … then those teachers should take classes to their students as a separate topic. If not for long hours, more often in small durations. If they conduct classes, then these children can teach their family. In this way programme implementation can be good." Health coordinator, SSI 20<br><br>**Some stakeholders said that mass media could be used to deliver messages:**<br><br>"… you need social media, you need obviously other campaign style information and messaging that gets out there so that when parents do for example hear messaging through the radio or on social media or on television or through their community networks they go, "oh yeah they resonates with me, because I heard the community health worker the nurse was telling me about that yesterday or last week or the last month" and reinforces those messages." National stakeholder, SSI 27
**Caregivers and health workers said that everyone should be treated the same and that the health facility should inform everyone about the programme to prevent conflicts in the community.**<br><br>"If everyone won't come to know about the programme, then there will be an environment of the fight. If we give something to one child and not to the other, there will be a problem. Similarly, these problems can happen … If all the children below three years of age are treated equally, then there will not be an issue." Mother, SSI 10
**Sub-theme 2.2 Focus on mothers, with the involvement of other family members**
**Caregivers, health workers and FCHVs said that fathers are busy and usually away for a job to earn money and will not participate in childcare.**

Participant’s responses
“I am not involved much in this … ladies are involved in these works. Whenever a child is asked for a visit, then she (his wife) takes him with her to health post … I am not available all the time, I have my job.” Father, SSI 1<br><br>“A father needs to learn as well, but in most houses the father of the children in Nepal mostly goes for overseas employment. So, there is no chance for fathers to attend such programmes for three years.” Father, SSI 3<br><br>“I think that even if you will attract males, then also, they will not participate. I mean to say that they will not be able to give more time. For example, a male who is working, needs to rush for the office on time at 7-8 am in the morning whether they have eaten food or not. But we are idle, stay in the home and even after managing household chores, we can manage time for the programme.” Mother, SSI 10
**Sub-theme 2.3 Participants’ expectations of incentives should be addressed**
**Caregivers indicated they would not participate in an intervention that did not include incentives.**<br><br>“If I am getting any facility, then only I will go; otherwise, why would I waste my time? Everyone does not have leisure time to spend for free (for nothing in return) … When people are tempted then only they will go.” Mother, SSI 10<br><br>“Whenever people are being called in any programme, they seek bonus, incentive, and lunch. FCHVs have been facing this issue in the community… people say to them, “You get allowance for this work, but we will have to come for free... why should we come?” so it is difficult to collect people …”, Health coordinator, SSI 20
**Health service providers and HFOMC members said that community people do not trust health workers if they are not incentivised and will not participate in the programme.**<br><br>“When there are new programmes in the community, there will be few percent of local people who will take in a negative way. They will question, “Who brought this programme? They (mayors, ward representatives or health workers) must have brought it for their own benefit. May be the focal person got lots of money. They get the allowances so they must have come to show us. Why should we take our children. My child gets scared. They can get scared.” These kinds of negative thinking could be there. When such challenges appear, everyone (local leaders, health post staff ward representatives) must support to take the programme ahead.” HFOMC members, FGD 3<br><br>“When doing programmes in the community like mass education … some can say, “they (health workers) have come for their own interest, they have come for their work, they have come to earn their salary. It is okay to not go there. Why should we go?”. Some people will have such negative feelings.” Health coordinator, SSI 21
**Some health workers, stakeholders, and a policymaker said that incentivising community members for participation in the programme has spoilt their habit.**<br><br>“Incentivising participants has a huge impact on us because before NGO’s (Non- Governmental Organisations)/INGO’s provided 200 rupees 400 rupees allowances whenever they came for a

**Participant's responses**
programme, which has developed a habit of receiving incentives in community members. We are still facing that problem. Even FCHVs also face this problem from the community. For instance, during Golden Thousand Days ("*Sunaulo Hajar Din*") people would get free snacks and allowance. So, it did not matter to them whether the programme happened or not, they would just do their attendance (sign the register) and leave as soon as possible after getting incentive. Now, whenever there is any programme, they start comparing "We used to get money like 200 rupees in that programme. Maybe this programme does not have any provision?". Somehow if they come to know if there is no money incentive then they will not go to participate in the programme." Health coordinator, SSI 20<br><br>"Problem is that many organisations have spoilt habit of people in many places. In the sense to make their project or programme successful or to increase enrolment they began incentivizing people without setting any norms. Now the beneficiary has developed a habit instead of understanding the importance of that programme they saw more value of money. Beneficiaries have been spoilt like this. Incentive should be promoted in case where in any place to promote any behaviour. For example, if they get incentive for check up in a health post or delivery in a health post then I would promote that because it will increase institutional delivery. The value of benefit is much higher than negative side effect. At the same time, if I interview someone and then I give them 1000 or 500 rupees or if someone say something to me and if I promote those things then I will get incentive, these will have more negative effects." National stakeholder, SSI 29<br><br>"… if users' group is looking for incentive, that is difficult, and we have spoilt the system because we have seen NGO's have been providing 200-400 rupees to them for attending a meeting for an hour. This is where things went wrong. We have seen this and experienced it. In one project, when we conduct a meeting for an hour, they (participants) asked us for money. In that locality, if they work for a whole day, it is difficult for them to earn 200 rupees. But we give them 400 rupees for attending a meeting. So, we are spoiling them. If that project does not go there, then they will be suffering as they will not get benefits. Participants do not see the long-term benefit. Instead, they seek for allowance for attending meetings this has been spoiling the situation." National stakeholder, SSI 30
**A stakeholder said that all the programmes should enter the community through the local government, let the local bodies make the decisions, so that there is no ambiguity in what people are receiving.**<br><br>"I: What can we do then? P: We should not take any programme from the outside. Now we should not do this. Let the local people under the local government take this decision. Right now, this is the problem. There is one programme from Plan Nepal, another from Save the Children and another from JICA (Japan International Corporation Agency). Some are giving this, and some are giving that. We are spoiling community people. I: They should go through the local government? P: Programme should be owned by the local government ... Let them take decisions. We (experts from external organisations) should give ideas and technical support only. If they need training, we can give them training for four days. But we need to leave all responsibilities of organisation the programme everything to local governments." National stakeholder, SSI 30
**Some stakeholders and a policymaker said that if the programme is delivered through the existing programme, then parents will not need to be incentivised for participation.**<br><br>"By mapping all the potential entry points within current service provision under the different sectors and maximising those, if the service is provided within the existing points through the health post, people will not ask for remuneration, which can also promote participation. When we are not asking

Participant's responses
people to make an extra effort but utilise the time, they use to reach the health post, then they will not ask for remuneration for participating in the programme." National Stakeholder, SSI 27<br><br>"If parents come to participate, they would not like to go empty-handed ... So, it would be better to integrate the programme with the ongoing ones. They will come with the desire to receive a vaccination, Vitamin A, or Baal vita (micronutrient supplementation). This way, our health and nutrition programme will be successful, and the play programme will go smoothly." HFOMC members, FGD 3
**Sub-theme 2.4 Involving health workers and FCHVs in delivery**
**Caregivers, health service providers, stakeholders and policymakers preferred FCHVs to deliver the programme to the community because they are from the same neighbourhood, they are familiar with the context, trusted by everyone.**<br><br>"FCHV knows everyone over here. She will roam around the village and even she is known to everyone. If the programme will be operated through them, people will go to participate immediately. But if a new person will be appointed to convey messages, then 50 % of the people will not take him seriously … and may ask questions like "Who is that person? We do not know him." … but if people from the health post will come people will feel "Oh she is from health post and is sent to visit house ... but if anyone sends through government then it will be better because people have already received services from those people and so they trust them. Nowadays, it's all about trust especially when it comes to children. Without trust, no one will send their kids." Father, SSI 3<br><br>"I: Why should female volunteers go for this work? P1: Because as much FCHV knows, villagers do not know about programmes. P6: We have taken every training. We know everything, therefore. I: And you sisters why should female volunteers do this programme? P7: See, female volunteers have been working here since long time. They have been teaching people about various things. We know about nutrition in detail. We do not have knowledge about games till now but now they have certain idea. […] P4: Yeah, female volunteers have taken trainings. They can do it better. P1: FCHV knows everything. They have knowledge of their ward. How many children are there? How many females are there? How many are mothers? and, in whose home, there are weak children they know everything." FCHVs, FGD 1<br><br>"Not just in our rural municipality but FCHVs are considered as the spinal cord of health system all over Nepal. Be it in any national programme or in any national campaign the programme will not be successful until FCHVs are mobilised. Staff from Government of Nepal do not have direct contact with the public community. They cannot interact with everyone. If they run the programme, then the programme may not be successful. They will not know information of each household … But FCHVs knows this very well. Number of houses in their work area, which house is having delivery, which house has a pregnant woman, which house has breastfeeding children, which house has elderly, which house has children below one year ... That is why it is difficult to run any programme if we bypass FCHVs. The role of FCHVs is indispensable." Health coordinator, SSI 21
**Some health workers, stakeholders and policymakers said that old FCHV do not want to retire. A health worker and a national stakeholder mentioned that the policy should be enforced. A national stakeholder suggested that the retired FCHVs could mentor the new recruited FCHVs, so they can guide and support the young FCHVs.**

Participant's responses
"In the beginning, to run the programme by the Nepal government, they recruited interested ones from villages as FCHVs. They are not much educated. They don't know how to write their name as well. Compared to before now every day the workload of FCHVs is increasing, their responsibilities are increasing. They must distribute medicines, participate in meetings. Majority of them have reached the age of around 60. According to the current time, at least those who have completed 10 class should be selected as FCHV who can recognise the name of medicines. They should be able to read aloud any written pamphlets and explain it to others. Those who are below 60 years, 40-45 years old FCHVs they should be rewarded and should be given retirement and, in that place, educated females should be made FCHVs. In this way slowly we can make FCHVs competent." Health assistant, SSI 22<br><br>"It is clear in the strategy that their retirement age, when FCHV reaches the retirement age she should transition to retirement … I hear as feedback that a lot of them don't want to. A lot of them are reluctant to relinquish their role to a younger woman. And so, the policy is not being enforced. There is a provision for that turnover and for the renewal of young woman coming in, but it is not just reinforced. I don't know about creating a different level of staff and trying to get around that ... just enforce the policy you have. Enforce it so that there is a turnover […] May be provide the elderly FCHVs with a mentoring role. Saying, "Okay you can mentor this new outcoming FCHV." which does not require elderly FCHV to be so hands on with all their activities, but she is there to guide and support the younger, the new FCHVs who has taken on the role. Again, it depends on how willing some of the women are to do that. It is a big shift they lose their status; they lose their position is their community and it must be hard." National stakeholder, SSI 27<br><br>"FCHVs will never say that their workload has increased and that they will not work. They will always say, "We will do it. We have been doing this for a very long time." because they have understood their face value and reputation in the community." National stakeholder, SSI 29
**Some health workers and stakeholders said that the FCHV are overburdened with responsibilities and they capacity must be considered when assigning new task to them.**<br><br>**"**Human resource wise we must think about how much work should be allocated to FCHVs. They are already volunteers. Like they have been regulating women's group. They have been doing other programmes like vaccination as well. Their training manual is of 200 to 320 pages. So, we give them so many activities and then if we involve them in stimulation activities too then we need to think whether they might be extremely overwhelmed as well. It would not have been a big deal if we were paying them money. Since our human resource is volunteer based, it would be a challenging factor if we hand over this stimulation intervention activities to them." National stakeholder, SSI 28<br><br>**"**Everyone knows the importance of FCHV in the community because of that everyone wants to use and mobilize them. At the same time, we need to consider about the quality as well. If we engage them in many places and then whether they were able to deliver quality of work or not or whether they could do or not. We need to think about this was well. In these terms we also need to be cautious. […] Health workers also promote their local FCHV. They never promote others. If we ask any local government, they will also want FCHV as they have been represented more. They have more value as they have been represented more ... If we consider education as a priority, we might get different thoughts and perception because many know that FCHVs are overburdened. To say they have almost become like paramedics. They diagnose pneumonia and give oral rehydration solution as well, they measure MUAC (mid-upper arm circumference) as well. If you look at their reporting format it has become dense than before. Majority of the register filling is done by their husband, if not then their daughter-in-law or grandchildren." National stakeholder, SSI 29

Participant's responses
**Some stakeholders said that hiring new staff can cause conflict and add financial load.**<br><br>"P1: … If we would hire new volunteers, they should be trained for few days whereas if we make the existing FCHV do the work then only a one-day information session is needed. New ones may not agree to do in same allowance. This can add financial load. The investment will be less when old FCHVs are used, and our programme will also be effective ... they are like staff from health post. There might be an issue if we hire someone else ..." HFOMC members, FGD 3<br><br>"… but to do through the government having an alternative category might not be beneficial, I think when alternative staff are recruited then there will be conflict about taking away their roles. Like I said earlier, stimulation does not take a whole day, it can be delivered together with other activities. It won't be cost effective if we add people for a half day work. I don't think we need to look for an alternative staff for stimulation in the current context." National stakeholder, SSI 28
**A policymaker said that government is planning to introduce ANM (Auxiliary Nurse Midwives) to the system and delegate clinical task to ANM and FCHV work will remain volunteers.**<br><br>"Since, in our country there are more ANM than FCHVs, we have been focusing on community based ANM concept too. We have made a motherhood road map documentation till 2030. This is a 10-year plan till 2030. We have introduced community based ANM in this. To improve the quality of services provided by health facilities, we need to add ANM in health post as well instead of just FCHVs. In coming days, we will be mobilising FCHVs as volunteers and ANM will be used to deliver the current health services and education […] To improve the quality services given by FCHVs in the community, health education is not sufficient. For this we are considering ANM. The main vision is that if any health-related projects are run via ANM in every ward, then the sole role of FCHV will be volunteering only." Policymaker, SSI 25
**Sub-theme 2.5 Provide interventions locally with a dedicated space to improve access**
**A health worker and a district stakeholder said that the programme should be delivered in the local language and by local individuals to encourage community members participation.**<br><br>"Whatever materials you will bring in this programme it should be in local language. All the staff should be local people. When community people see this; they will feel easy to come to the programme. Not the outsider but a person from the own village. It could be someone's sister-in-law, aunty (*kakai, maiju*) et cetera. People will trust saying, "they have come for a good work. They are not outsiders. They will not hide anything. They will not take any pictures to put on the social media or internet." They will have that kind of trust and will be interested to come to the programme." Health assistant, SSI 22<br><br>"… We need volunteers from local level. If third party is going to work, generally, first community wants to know about the people and the name of the organisation those people work for. For instance, whenever trainers from [our organisation] comes in this village, people get excited and attend the trainings because they recognise our staff and trust them. But if some other outsider visit, then they question, "Who are they? Why are they here?" They don't get involved with new organisations. We need some trustworthy and familiar people." District stakeholder, SSI 23
**Some caregivers, health service providers and HFOMC members said that lack of physical facilitates for the programme can discourage community members participation.**

Participant's responses
"In my village, there should be a small place for children to play and any materials to play with should be provided because a child under three years of age starts to play and, at that age, they will learn ABCD, or they will play. They will learn through games. If that could happen, it would be good because a child under three years won't go to school to study." Father, SSI 3<br><br>"I: What will facilitate the community to learn from FCHVs? P: Firstly, there is no place to sit in our community. There should be an environment for a group of 10-20 member to sit. There could be a lactating mother. Because we are talking about mothers of children below three years age and many mothers with small children breastfeed their child. There is no place to sit and give counselling to those mothers. There is no private place. It is uncomfortable to cover yourself with clothes when males are around. It is difficult for mothers to breastfeed in the open area … In our community we need physical infrastructure first […] If not more we must give tents to FCHVs, for health workers in every outreach clinic and immunization clinic we must give two chairs, one bench and one stole. These things must be compulsorily available for them. We have seen their problem. They do have a problem." Health coordinator, SSI 20<br><br>"P1- … Children make that place dirty right away. We do need bucket, soap (with laugh). Don't we need that? Is that not a problem? We need soap, broom, sanitizer, face mask. If these are all available, it would have helped us madam. P3: Sister if all these are managed it would have been easier." FCHVs, FGD 2<br><br>"P1: Open space is not preferable for teaching mother and their children … In Terai (lowland), during the rainy season, there would be cold waves. Carpets is needed so that could be used to sit on. The local government or all three level of governments should help in this." HFOMC members, FGD 4
**Sub-theme 2.6 Health posts or outreach clinics in communities are good locales for delivery**
**Participants suggested using a comprehensive approach to deliver the stimulation programme.**<br><br>**Health service providers, stakeholders and policymakers said that the stimulation programme could be delivered through the existing contact points in communities:**<br><br>"First is in the outreach clinic mothers can directly come with their children and health workers can do the counselling activities with them. In such case, we can counsel mothers about how they should behave with their children. Mothers can be counselled individually […] Another is when they visit mother group meetings. Counselling can be done in the clinic but to reach the community we must go to the mothers' groups." Health coordinator, SSI 20<br><br>"…when a parent brings a child to the health facilities or the health post for another service, that health worker can have, even if for just a two-minute, a conversation with caregivers about the role early stimulation can play in helping in the further development of a child and maybe childhood development milestones." National stakeholder, SSI 27<br><br>"If you map the contact points you have with parents. What are those contact points? When a mother is pregnant is a contact point, when the woman delivers there is a contact point, when she comes back for various child health services like immunisation, growth monitoring, there are a contact points, when she first enrolls the child in kinder garden there is a contact point ... If you look at the contact point along the continuum or the life cycle of the child and identify what are the services, you can embed and

Participant's responses
integrate early stimulation and ECD into those … By mapping all the potential entry points that exist within current service provision under the different sectors and maximizing those because if it is not embedded into particularly government system and government service delivery points you risk paying lots if it just led by NGO or INGO or external agency. You risk it only being sustained through the duration of that support once that support is gone, the programme is gone […] I think that the health post has opportunities to demonstrate the stimulation and what it is, by having spaces available for children to play, showing mother caregivers what sorts of things they can do at home, what it really looks like … it is important that they talk about how, not just what … how to do it, how to overcome obstacles, who can be involved, who should be involved, what role can the broader community play, so those things. But health post can demonstrate, they can have physical spaces … they have like nutrition corners in some of these public spaces." National stakeholder, SSI 27<br><br>**Caregivers, health service providers, stakeholders and a policymaker said that the programme delivered through the mothers' groups can reach the wider community:**<br><br>"M: What are your reasons to choose mothers' groups? P: My reason is that in health sector mother's group is our team. Like FCHVs are there, mothers' groups are one of the team of health sector. We go through this team to do any programme. First, female health post staff and then FCHVs and then through mothers' groups we reach the community. When we go to the mothers' group if even one person out of 20 is motivated then that is more than enough. The objective of mothers' groups is to teach others in the community what they have learnt in the group. If 20 mothers from mothers' groups can teach at least one person in the community about what they have learnt, then second, third, then many people will be included. If that happens then there will be chances of very few people to get left out from the programme. This is our team so we can follow up them time to time to ask if they have shared any information to people." Health coordinator, SSI 21<br><br>**Policymakers said that groups are not functional everywhere:**<br><br>"Pregnant women groups, mothers' groups, mother-in-law and father-in-law and husband and wife. These are the 4 groups currently in the policy level. The thing is that these groups are not functional in every place. This is the problem .... I: You said that it is not functioning, why do you think so? P: We have done special budgeting for all these groups only in MIYCN programme and it is in operation as well. The programme keeps on operating until they receive budget. If the budget stops, then the meeting in that group also stops. They expect snack, allowance in every programme related to INGO/NGO. They do not get allowances, but snacks in any governmental programme. In MIYCN, we have been sending the budget for the mother's group and mother-in-law and father-in-law group, so the group are being regulated. Through, family planning programme, husband and wife are getting allowances, but the budget has not reached every municipality. So, this may not be regulating in all places. Initially, all ward received budget, so it was functional everywhere. But now programme is operational in those places which receive budget, otherwise they are not functional." Policymaker, SSI 25<br><br>**Most caregivers, health service providers and HFOMC members said that home visit is not feasible:**<br><br>"P: … because it is not possible to follow door-to-door visit. I: Why do you not find door-to-door visit possible? P: Because it is hard to manage time for every house. It will take more time and so in just one village it will take nearly one month. If she (FCHV) will follow door to door visit and in one locality there might be 20 houses and in 20 houses it is not be sure that they will find 20 children. There might

Participant's responses
be 10 children only. So, if those 10 children can be brought together and taught to play, they will be taught in a single time. It will benefit them all in a small time" Father, SSI 3<br><br>"I: You did not select the option of a home visit. Why? P1: If we do a home visit, we must give more time […] P2: In the house, they have their work […] they will think of their work and will not listen to us. P6: If I visit their house, they will say they have to do their work and will not give time. P1: … How many houses can we go to if we visit each one? It will take more time." FCHVs, FGD 1<br><br>**Caregivers from marginalized group preferred home visits:**<br><br>"I: In which way should she (FCHV) teach you so that you will like, and it will be beneficial for your children, for you, for everyone else. P: For us, she should visit our home. M: Home? P: Yes, if she will come to my home, I am the guardian and I have my grandson and granddaughters. If in case I am not available at home at that moment my daughter-in-law will be available, FCHV can teach her as well and when I return my daughter-in-law will tell me. If they (FCHVs) call us in a meeting, sometimes I might not go, and my daughter-in-law does not come out of the house. So, if I will not go then I will not be able to know what happened in the discussion. But if she (FCHV) will come to our house than my daughter-in-law will come to know and when I will come from my farm or from any other place, she will tell me." Grandmother, SSI 15
**Health service providers and HFOMC members highlighted using multiple activities that could be used to teach community members about stimulation.**<br><br>**Audio-visual and pictures:**<br><br>"M: What method should be used such that villagers can understand easily? P2: … By using the method of video and audio. For video, it can be done through television. M: Through television! P1: It is easier to do it using a projector. At a suitable time, it can be shown to the parents of children. M: In what way should volunteers teach such that it is easier for the mothers in the village? P3: Pictures can also be used. It can also be done with videos. Only these two methods could be used and there is no other method. Either videos or photographs. M: Why are you giving importance to videos and photographs? P3: The reason why I give it more importance because there is a lack of educated people. Majority of people are uneducated. They can understand by looking at the pictures. If it is not available, they can look at videos. Therefore, at least one should be available. It is a good thing if both are available." HFOMC members, FGD 3<br><br>"I: In which way should the programme run in community so that it will have an impact in your village? P: In community by making groups and using pamphlets, posters, and flip chart. I: Why do you think these things will be effective? P: Because just by seeing, public will feel the pictures. They will try to follow the pictures. They will feel like if they do according to the pictures it will help in their children growth and development." ANM, SSI 19<br><br>**Demonstration methods to teach playing local games using local toys.**<br><br>"In the community where the programme will be done … make them practice minimum play and learning related activities with children, identify the need of play materials that are locally available. Cheap, easily available and lasting play materials must be made available and bring them into use. Toys that are less costly, fine, attractive and influential must be selected. At a local level we can find "Ghirni" made from bamboo by people from Mallik (Dome - Dalit) caste, which is a colourful toy.

Participant's responses
When we use such toys, it will attract children as well as it will look like those communities are being fostered […] Demonstration method would be fine. Like how we used to teach steps to prepare oral rehydration solution. In similar manner put 2-3 types of toys in front of the child and show them each toy one after another and play with them." Health assistant, SSI 22<br><br>"Local games should be included, not the foreign-based games, which parents cannot afford to manage tomorrow. What can be done in the local context that should be taught, then they will be able to practice at home. They will be able to manage toys. We need to capacitate in that manner." District stakeholder, SSI 23
**Sub-theme 2.7 Strengthening monitoring and evaluation strategies**
**Health service providers, stakeholders and policymakers reported that the monitoring mechanism should be enhanced through a skilled workforce. Health workers must be updated on knowledge and skills on monitoring tools and techniques through orientations and adequate trainings.**<br><br>"Before co-facilitator sisters were MCHWs (Maternal and Child Health Workers) and are now upgraded as ANM. But they have only studied to 8th standard and if they don't have knowledge and attitude about health services then it will be difficult to provide those services ... An upgrade is needed for health workers through programme orientations and trainings […] Many health information system tools have been changed by the government once our country changed to secular state. Trainings have been provided but, the way these trainings were provided was not fair. Like 100 people were given trainings at a time. What will 100 people learn? They received allowances. But they even don't know what has been listed in the integrated child card. I think those trainings process was not that good. We should not tell everything at once to the health workers. They may not be able to take up everything at once." Health coordinator, SSI 20<br><br>"I: So, you said about lack of monitoring resources, right? What else did you find in health post? P: … Government staff are also not capacitated (trained enough). Government also don't have the capacity in terms of their staff as well. Public service commission has the potential, but they lack capacity programmatic wise. I: Can you give any example? P: For instance, if you take an example of any health worker who has completed his/her education up to IA (10+2), they are from non-health background and their work will be related to health background. They cannot monitor anything related to health background. How can he find about SAM (Severe Acute Malnutrition) or MAM (Moderate Acute Malnutrition) cases? He does not know how to identify stunting. Non-health background person cannot go beyond what he was taught in the orientation. This will cause a big problem […] Governmental staff find easy to do easier task and difficult to do the advanced tasks. They find it difficult because they don't adequate knowledge … we don't have health workers who has adequate knowledge. If that person does not know something, then he won't be able to speak or perform. It is not a big deal to fill the growth chart, they come and measure the weight and then when it comes to filling the level the child falls in the form, they cannot even find the arrow. They will write anywhere they like. Suppose if he must fill weight of 7.5 kg, then all he does is note down in 12th line, which is already wrong. We don't have that kind of skilled people here." District stakeholder, SSI 23
**A few health workers and stakeholders said that new indicators for stimulation should be included in existing national and state-level surveys to improve transparency and accountability in programme delivery.**

**Participant's responses**
"We have a reporting format in which the number of households in the green category, number of households in yellow category and number of households in red category are collected. This report comes from FCHVs. They visit the community and observe each case. When the stimulation programme is combined new indicators could be added to this format. So FCHV can collect this additional information too along with the nutritional related information. Along with the nourished children, how many families play with their children; how many households do not have toys. We could also use colour coding. How many households in green category were children played with. How many houses within the yellow category children were played with or have toys? How many households do not have toys? Similar questions can be added to the reporting form, do the reporting of the programme implementation, and identify children who were undernourished and who were not played with." Health coordinator, SSI 21<br><br>"It comes down to how do you build the indicators and what indicators do you build into information systems and data recording. FCHV must record whether they have had some sessions with parents on early simulations, how many households for example, they have been trained or involved etc. Then they start to be more accountability for that. Same with health workers. […] At present there is no indicator in DHS (Demographic Health Survey) on ECD. So, there is an opportunity there to identify, get an agreement or consensus on what could be included, which indicator could be included and then of course that means having to revise the health facility registers, which is the starting point for data collection and reporting and recording of services provided." National stakeholder, SSI 27
**Some caregivers noted that different castes would not mix and may feel conflicted about having a separate intervention for their caste.**<br><br>"For children, people from different castes may fight, saying, "I will open a different group for my child, or you should open a different group for your child." That might happen. They may fight, saying, "I will not let my children participate in this." Like I am a "Sahu" [landlord], and I will not allow "Doom" [Dalits] children to play with mine. I will not let my children play with Muslim kids." Father, SSI 6
**Sub-theme 2.8 Mobilise local opinion leaders to overcome religious and caste discrimination**
**Health workers and stakeholders said that the marginalised groups must be approached through their leaders.**<br><br>"Within the community they have leader… we should call the leaders of Dalit community and Muslim community as well. Community people will listen to their leaders… Must involve Mullah from the Muslim community … they have strong leadership (eka hukum) ... whatever a Mullah (Maulvi) says that will be implemented but if they don't say, nothing will happen … the involvement of disadvantaged (pachadiyeko) community must be compulsory." Health coordinator, SSI 20
**Engage household heads and officials to encourage the participation of young women**
**Health service providers and district stakeholders describes that the newly wed brides and a new mother are not allowed to leave their house, however if the guardian understands the importance of the programme, they may give permission for participation.**<br><br>"A mother with a child less than three years might want to attend the programme, but if her guardian does not allow it, then she won't be able to do. That is why everybody plays a role. That is why all guardians must be well motivated. One member in the house may be interested, but if the house owner

Participant’s responses
(household head) is not interested, then everyone will be deprived of the knowledge.” Health coordinator, SSI 21
**Most caregivers, health service providers and HFOMC members said support from the community representative and leaders is must to gain people’ trust and encourage participation.**<br><br>“It will be good to explain about the programme in the presence of representatives such as teachers, health workers, FCHVs, educated women, ward members. They should be included in the orientation. Recently, all programmes are looked from the political perspective as well whether that person was present or not. […] Like there are politicians, mayor and other authorised officials. When they come to the village for supervision, when they talk or speak about such programme even for five minutes saying that this programme will improve child growth and development, child nutrition will improve then it will have a good effect on the community. Community people will understand that the programme must be good that is why these community leaders are talking about it.” Health assistant, SSI 22
**Sub-theme 2.9 Support poor households to ensure their participation**
**Some caregivers, FCHVs and stakeholders said that poor family cannot afford play over food.**<br><br>“[…] if I am unemployed and have no job, my house will run only after I earn some money. So, earning is necessary. For instance, I am educating my child, although I don’t have that status. Still, I want my child to get educated. But if I won’t have that much money, how will I educate them and so I must work ... If I don’t work, things will not work for me ... So, I will not be able to manage my time like whether go to job or those programmes.” Father, SSI 1<br><br>“Some kids in this village belong to poor households. People from these households do not have enough to feed themselves. So how will they teach their children. There are kids of age six months, one year, three years. If children are hungry most of the time, how can we educate them. One parent goes to the field, and another goes for labor work. They leave their children at neighbours. Only a little amount (of money) comes from labor work! So, should he provide education for his kids or fill his belly?” HFOMC members, FGD 4
**THEME 3: Support from national and local governments**
**Sub-theme 3.1 Prioritise funding for ECD at the national level**
**Some health service providers, stakeholders and policymakers said that the federal or local government does not prioritise investment on ECD.**<br><br>“… according to the people’s representatives (the focal person in any development activities in the community), development does not mean the health sector. Health is not related to development. According to them, infrastructure, roads and irrigation are considered development factors. For instance, we had a massive cut in the budget allocated for the health sector only. […] They do not consider child development an important subject ...” Health coordinator, SSI 20<br><br>“The magnitude of this problem is bigger in the community and the amount of attention that it should be getting, the amount of share that it should receive, it has not received enough share. If you see, we only have 4% of the budget for health care. This year I think they made it to 5%. According to the World Health Organisation, budget should be at least 10% to address the prevailing problem. We can

Participant's responses
see this. Even research shows that the government should spend 10% of the budget on its health sector otherwise the problem won't be solved especially in this underdeveloped and under privileged society." Policymaker, SSI 24<br><br>"We do a budget analysis with the Finance ministry at the beginning of any new programme. It becomes easy when we do budget analysis based on the difference. After running the programme for 2-3 years, finance ministry does not approve to continue the budget. They say, "This is community level work, and we don't need to spend money on allowance and food." Their approach is that volunteers should run this programme free of cost. It is not easy to run programme like this in community. They start cutting the budget by 10%, then 20% and we are forced to stop the programme in certain district. The places which don't have any more budget the programme discontinues." Policymaker, SSI 25<br><br>"Finance ministry always have budget related problems when we talk about programme for ECD. They say that they cannot do it. One is the financial reason. In our country the budget for the development in the last 40 years, 80% of our country's budget is used in construction work like roads, electricity, and bridges. We talked to the secretary of NPC when we were working on the ECD guidelines. They said that 80% is allocated for construction and remaining 20% is utilized by the other aspects. Certain budget is allocated to the education and within that less than 2% is allocated for ECD. One side they do this and other side we talk about holistic development and ECD so there is a problem financially in terms of political commitment … we lack political commitment because of vested interest because the return in result is slow so political leaders and local government don't want to directly invest in ECD […] Even the mayors from local government say, "this is a nice thing but if we spend more on infrastructure development then we will get higher votes." We have this kind of problems." National stakeholder, SSI 30
**A few stakeholders and a policymaker said that evidence should be generated to advocate the government what works and how much budget should be allocated. Budget allocation must be supported with evidence for approval.**<br><br>"… we need a well justified document. This will help us deal with the Ministry of Health and Ministry of Finance for budget allocation. We will be able to deal with them. We will be able to explain them and then the budget will be allocated […] For budget allocation in central level, we need to have well-documented evidence showing that this stimulation intervention works. We need to have evidence for budget allocation and the process will be easy. Without documentation, budget allocation is going to be difficult. If we want to implement some new interventions or a new programme, we must have evidence or we must have a result report of a piloting programme. Piloting programme can be done in any small district. We need to do piloting first then document and provide evidence-based documents." Policymaker, SSI 25<br><br>"The resources that are available I don't think are being used efficiently as they should be. The budget allocations are not always evidence based. The decision on where and how to allocate funding, to which programmes is not always taken into the account the priority needs of the programmes or the situation on the ground. So, I think there is a lot more work that needs to be done to build capacity for result-based planning and budgeting." National stakeholder, SSI 27<br><br>"… Until we do not make a fund-raising proposal for integrated programme and until we do get a proper funding, till then we cannot run any programme in full phase. […] They have the budget, but they have not understood its importance. In this situation how can we an investment case. If we can make an investment case and tell them that if they invest in this, then this is the benefit to our children

Participant's responses
and when those children benefit the society will benefit. In this way we can show them (Government of Nepal) [...] If we can show this kind of evidence through research and make an investment case then government funding can be ensured." National stakeholder, SSI 28
**National stakeholders said that ministries could jointly allocate funds for integrated interventions at the national level.**<br>"… if we want to take the ECD and nutrition activities at the community level, then we can also manage joint financing. That means we need to ask the Ministry of Education how much they can invest because they can also invest from the national level. Similarly, how much investment can the Ministry of Health make because we might also take some components through FCHVs." National stakeholder, SSI 29
**Sub-theme 3.2 Recruiting new health workers**
**A few health workers, stakeholders and a policymaker stressed on increasing the qualification criteria for health workers and recruiting a workforce with health backgrounds.**<br><br>"If you take an example of any health worker, who has completed his/her education up to IA (10+2), they are from non-health background and their work will be related to health background. They cannot monitor anything related to health background. How can he find about SAM or MAM cases? He does not know how to identify stunting. Non-health background person cannot go beyond what he was taught in the orientation. This will cause a big problem." District stakeholder, SSI 23<br><br>"… health workers from health post do not have the capacity to fully know about stimulation. They even do not know about the linkage between health and disease. Where and how? This internal aspect of public health, this epidemiological linkage and many things are not known by the local health workers. They don't know these linkages. They are underqualified ...That is why we should hire public health graduates in municipality's health post now." Policymaker, SSI 24<br><br>"The current health workers in the health post were appointed based on the workload from the previous 30 years. In the current situation, we cannot function unless we hire new health workers. It seems like we need twice as many as we currently have. For this, provincial and local governments have hired temporary workers to help with this. They have been hiring 4-5 staff recently." Policymaker, SSI 25
**Sub-theme 3.3 Review training, supervision and incentives for health service providers**
**A national stakeholder stressed that people who will deliver the programme at the field should be trained.**<br><br>"There needs to be some sort of shift in again thinking and delivery of capacity development for the different stakeholders. On the job training needs to be I think more emphasised rather than training district health coordinators. They are an administrator they are not in service delivery role ... So, I have always argued, "oh why you are teaching them to how to use a MUAC tape". Because they are never going to use a MUAC tape in their life. The health workers are going to use a MUAC tape. FCHVs are going to MUAC tape. So, train and master a pool of trainees of health workers don't train district health coordinators." National stakeholder, SSI 27
**A stakeholder and policymaker said that the high turnover of master trainers, makes the training process costly.**

**Participant's responses**
"Whenever we start new component, we find it challenging to find trainer who is the right person and responsible. It is a challenge to mobilize the trained trainer once we have provided training. Suppose there are 21 people who completed master trainers, only 10-11 trainers are left for mobilisation. Some get transferred, some have gone out of the system, … or if they are medical doctors (MBBS) they could have gone to study MD after the training. They could have taken study leave. Those challenges are there in our system. But even if they are in the system, sometimes their organisation does not allow their leave for training upon our request. They won't be available for the training. So, the challenges are they could train but may not be available for the training when required." Policymaker, SSI 24
**Some stakeholders and policymakers said that training courses are crowded and short, hindering participants' ability to understand and retain information.**<br><br>"… we provide training to FCHVs for two days to orient them. They learn and go to the field. They can get confused in the field because more than 20-25 people had participated in that training. Everyone has a different rate of understanding in those two days of training." Policymaker, SSI 25
**A policymaker highlighted that there is a difference in incentives by external agency and government, consequently, health workers are less motivated to do government- related task because their expectation of incentives is not met.**<br><br>"What we do here is that to do a piloting, we take up more resources than we need for small intervention in few places. This will make it look like successful. Once it reaches to the government, we need to follow the rules, regulations, and norms of the government. When we start following that, available resources will get depleted due to this. Our norms and donor's norms do not match. Similarly, UNICEF, GIZ, USAID and WHO norms does not match with ours either. Everyone has different norms for incentives. In this situation, it becomes challenging for us because when successful piloted programme is owned by the government, problem arises in its survival. The problem is due to nothing else but the allowances. Simply to describe this. WHO provides field allowance of 2500 rupees to a person while Nepal government gives 1600 rupees only, which is DSA (daily subsistence allowance). Hence, they (health workers and FCHVs) prioritize project activities more than that of governmental activities to receive more money […] Where we saw problem is government could not take over projects made successful by UNICEF and when government was able to take over the project there was no acceptance due to financial norms. It wouldn't be a big deal if they provide 400-800 rupees as allowance from the beginning. If an NGO pay 400 rupees same as government, then it doesn't make any difference to FCHVs. They will work with motivation, but it is difficult when they receive 1000 instead of 400 rupees for the same work. This is challenging and we are facing this internally in our system as well. This has affected the programmes." Policymaker, SSI 25
**Sub-theme 3.4 Local governments play a key role**
**A few health workers, stakeholders and a policymaker have said that with the current political transition of Nepal from unitary to federal governance, implementing any programme at the community level has become challenging.**<br><br>"The implementation process is slow. Even now we have seen slow hiring of health workers and teachers as well. There is a lack of technical assistant in many places. It is taking more time to fulfill human resources. It won't be hard to implement ECD related activities if all these things are fulfilled. Many municipalities are also dependent on INGO and NGO for human resources as well ... Slow

**Participant's responses**
implementation of decentralization is the problem […] For full implementation of decentralization, power should be handed over to the local level and local government should also realise their power …We tell people from the municipality and when we tell them field visit is needed and tell them we need junior level technical assistant … This post has been vacant for last two years. Human resource allocation is done by internal affairs ministry. It would have been more effective if they have had hired that person timely. We see barriers like this at local level. So, the decentralization has not been completed. It is moving in a slow pace." National stakeholder, SSI 29
**A health coordinator stated that many staff in the municipality are newly appointed and muddled due to a lack of clarity on responsibilities.**<br><br>"Now talking about recent days, this village has been promoted to municipality in 2073/2074 B.S. In the beginning, mayor sir, officers … they all were newly appointed, and everyone found it difficult to understand the procedures and the regulations for 1-2 years. It seemed like it did run well in the beginning. But when observed the results ... like the work to measure the weight of children was not going in place. It was not going like how it should go. No pregnant mothers and children were motivated like they should be. Even if we see the data now, we find that it is lagging behind." Health coordinator, SSI 20
**Some stakeholders and policymakers said that local government do not know what policies there are at national level. The national government does not advocate and orient local government well for them to act on the national policies and strategies.**<br><br>"All the documents are prepared at the federal level because that is the job of the federal level. They make rules and regulations. If we see the penetration (dissemination) level, it has never reached the local government. Local government would not know that there are certain policies made related to a certain subject matter. That there are such strategies. That there is programme planning. They won't know. If you talk about MSNP. They call MSNP as a project. They (the federal level) have not talked about this at the local level. They have not said that MSNP is a plan and that this we have to take as an integrated manner. So, people at local government think that this is a project, and certain budget is allocated for this. After some time, the programme will finish. Because the penetration of information had not happened in a uniform manner from federal to the local government. I see this as a major problem." National stakeholder, SSI 29
**Stakeholders and policymakers said that local level does not have the capacity, but they are willing to learn and launch new programme which provides an opportunity for integration.**<br><br>"At local level there is no capacity that is the main thing. From start to the end, it should begin from the top level. Everyone should be made aware about ECD. The chairperson of the municipality should be made aware of this information at first. Until you don't lobby and convince the chairperson of the municipality, I don't see the possibility of this programme will move forward. After that the technical person from the administrative area should also be capacitated. They don't have the knowledge too. Stimulation is a new thing, which is taking place in foreign country only, but stimulation has not come much in our country. If we search in websites as well, we are not going to find anything related to our country as people have not worked a lot in this field for children below three years." District stakeholder, SSI 23<br><br>"What I had heard was "they (officials from local government) won't let us (representatives from provincial government) in, and they avoid us. That there is a denial". I heard all these things … I have

Participant’s responses
gone in more than 16 local bodies, and I have not seen any denial or refusals. They said, “Sir/Madam need to visit more … no one comes here, and no one gives any valuable feedbacks or trainings to us. No one support us …We don’t know anything is this subject matter.” Policymaker, SSI 24<br><br>“Whenever we reach out to them about any programmes and give them directions, municipality’s mayor, sub mayor and ward president say that we need to teach them first, they say “we are new on this”. The gap that they have identified in terms of capacity building, and that they tell us to teach them, provides us one opportunity that if we are able to encourage them and build their capacity, they could help us in reinforcement of our programme because they have the full authority for supervision and implementation.” Policymaker, SSI 25<br><br>“… that’s why we can use the platforms to raise awareness. So, taking the issue down to the ground, doing orientation sessions with a local government and saying, “Hey let’s teach you about early stimulation.” They are very receptive once they know about something. They want to learn; they are hungry to learn and once they know something, “okay this is relevant for us, we see the value in doing this ... this is worth investing in …” they do it. It is really encouraging to see how much the local governments do actually adopt and take up and some of them do it spontaneously on their own. Some of them require a bit of a nudge.” National stakeholder, SSI 27<br><br>“Local governments can plan and do what they want, but they do not have the technical expertise about what to do. They are looking for the federal government for their guidance … There is a need for a lot of capacity development of the local government to contextualise and adapt and turn the national guidelines and strategies into actions.” National stakeholder, SSI 27
**Some stakeholders and policymakers said that evidence from local context regarding what works and what does not is needed to support programme implementation.**<br><br>“We need survey, research in province based or district based, or country-based in this subject. We can seek help for this from NGO and INGO as well. Then in our socio-cultural context how do children behave? How do we behave with our children? What kind of stimulation we are providing? And what kind of behaviours do child develop? What is their thinking process? How is their perception developing? We can do that study here … We need to do research and survey at local level. This is an important area where we lack interventions. For Madhesh Pradesh, it should be done in Madhesh context. Then we will know what kind of stimulation should be provided, what local resources can be used to develop materials.” Policymaker, SSI 24
**Sub-theme 3.5 Governments can get support from external agencies**
**A few stakeholders and a policymaker said that it is difficult for the government to reach everywhere; support can be taken from the NGOs/INGOs and CBOs to support the local government.**<br><br>“Their presence is needed in the local level during implementation phase where federal level approach and supervision is less. Its better if they are greatly involved and provide help in community level. If I must take an example, there is a programme named SUAAHARA project, in which they have effective presence in the community level as they have supported in community level activities … This provides good quality assurance. I have seen due to their good presence in the community level activities in SUAAHARA, we are receiving good support.” Policymaker, SSI 25

Participant’s responses
“Since we have few health officers, we (the government) cannot attend every mother group meeting. Where NGOs/INGOs staff are present if they could support those FCHVs running the group sessions or while interacting with the group in any new interventions. Provide guidance and supervision to FCHVs and help them. Sometimes if wrong messages are being delivered, then that gets corrected. This makes it easier and is seen as a good thing.” Policymaker, SSI 25<br><br>“If there are any organisation to provide technical assistant in each municipality then it would be much more beneficial. We also tell in many workshops of NPC that the government cannot not reach every place for technical assistance. It is difficult for the organisations at federal level to reach everywhere to provide technical assistance. If we could strengthen Community-based organisation or NGO at the local level or if they already have capacity, then we must create an environment where they can support the local government. For example, let’s say if there is an NGO which works in education then they can provide technical support when they are working with the municipality. A person who is in municipality they are not health or nutrition expert. They are the general people but if the NGO working in that place is a health specialist or they work in education or ECD then they can do advocacy as well. They can provide technical assistant to the municipality.” National stakeholder, SSI 29
**A policymaker said that NGOs and INGOs should provide technical support in generating new evidence, but the programme should be delivered through the government.**<br><br>“NGOs/INGOs (should not intervene directly though) provides technical support in the beginning then; they can help maintain programmes quality. But it is impossible for them to stay forever in reality. The Government of Nepal or any government requirements is to see the effectiveness of a new intervention/programme before allocating the budget. We need help from supporting agencies for piloting a new programme. If everything seems successful after they have been implemented (by external agencies), then only government will continue ... It won’t be good if we also go parallel with regular programmes … After they have supported the programme implementation for a certain time, the best way would be to hand over the programme to the government and bring it under the routine programme.” Policymaker, SSI 25
