# Supplementary material for: Exploring the feasibility of integrating health, nutrition and stimulation interventions for children under three years in Nepal’s health system: A qualitative study
Source: PLOS Glob Public Health. 2023 Apr 28;3(4):e0001398. doi: 10.1371/journal.pgph.0001398 (PMC10146516; doi:10.1371/journal.pgph.0001398)
Supplement: S1 File — (PDF) [file pgph.0001398.s003.pdf]

S1 File: Topic guides for semi-structured interviews and focus group discussions

Semi-structured interview guide for mother/father/grandmother with a child less than three years of age

Date of interview: ……………………

Location of interview: ……………………

Interviews start time: ……………………

Participant number: …………………….

**Inclusion criteria:** Before beginning to confirm that the participant is from Maithili speaking population and a mother/father/grandmother of a child less than three years age.<br><br>**Consent:** Prior to beginning the interview, participants will be read the consent form and give written consent and an oral consent on an audio recording. Give the participant a copy of the consent form and the information sheet.<br><br>**Introduction:** Interviewed should explain the study purpose to the participant and answer any questions he/she might have.

Collect some information about the participant:

- Name:
- Age:
- Caste:
- Religion:
- Marital status:
- Highest level of education attained by mother:
- Highest level of education attained by father:
- What job does the mother do?
- What job does the father do?
- Main source of household income:
- Time to walk to the nearest health post:
- How long it takes to get to the hospital in Janakpur by the usual transport you have access to:
- Total number of children in the household:
- Age of the youngest child:
- The number of adults who live in the household most of the time:
- Number of children participant usually cares for:
- Do the child/children go to an ECD centre or another childcare centre?
- Contact number:

Instructions:
Use these questions to guide your conversation with the participants and probe where necessary. Statements in **bold** inform the participant about the topic of conversation. Indented questions are follow-up or probing questions.

Rapport building:

a. Can you tell me about your daily routine in looking after this child (under three years of age)? (Probe: Who looks after children? Where? How? Who feeds the child?)

Following this do activities one and two from the vignette with the participant and their youngest child (under three years of age).

Availability and experiences of stimulation (play and learning) services

Now I would like to ask about the play-related programmes (availability) at the health facility and in the community.

a. What do you think other people do to improve play and learning of their children?
b. What services/ programmes do you use in your community related to improving play and learning between you and your child?
c. What kind of support is there for play and learning for children in your local area? (For e.g., informal playgroup in the community)
d. In your view, what kind of support/ information is needed to improve play and learning-related practices?

Availability and experience of nutrition services

Now I would like to ask about the nutrition-related programmes (availability) at the health facility and in the community.

a. What nutrition services do you use in your local area?
    i. Probe for different kinds of programmes for mother and child nutrition (at the health facility or in the community, give examples of programmes for nutrition such as providing education about feeding your child, and measuring child's height and weight)
    ii. If they do not use any services probe for any nutrition programme, they are aware of.
b. What do you think about the services provided within those programmes?
    i. Probe the programmes they have mentioned.
    ii. What are your views about the care/information/advice given there, the staff who provide the services, and the activities they do?
c. What are the things that have made it easy for you to participate and follow the advice provided through nutrition programmes?
    i. What has made it difficult?

Integration of playing (stimulation) and nutrition services

Now I would like to ask you about improving play activities

The World Health Organization (WHO) has announced a new guideline to improve child development. The WHO recommends combined nutrition and stimulation/playing interventions for children below three years of age. (Give examples of programmes for nutrition and play/learning)

a. What are your opinions about adding play and learning-related activities to the current nutrition programme that is provided at health facilities/in the community?
b. What effect it might have on your children and other children of similar age in the community?
c. In your view, how would people in the community see the introduction of play activities along with a nutrition programme?
d. How do you think such a programme should be delivered?

   i. Where do you think the programme should be delivered? (At the community? At home? At health facility?)
   ii. Who do you think should deliver the programme?
      i. (What is the reason for choosing that health care or other provider?)
   iii. Who should participate other than yourself?
      i. (Probe the respondent themselves, and other family members. What are the reasons? How would their participation benefit in childcare?)
   iv. Who do you think should be involved in making changes (adding play/learning activities to the current nutrition programme) happen?
e. In your view, what could be done for the sustainability of the combined programme?
f. What challenges do you think there could be for the health system level to carry both activities related to nutrition as well as play/learning together at the community level? If not, why?
g. If play and learning-related activities were happened to be added to the current nutrition programme
   i. What are the things that might make it easy for you to participate in the programme?
   ii. What are the things that might make it easy for you to follow the advice provided through such programme?
   iii. What might make it difficult? (Probe for social and cultural barriers)
   iv. How would you like to contribute to the implementation of the programme? What could be your role?

`

Conclusion: Thank you very much for your time. Is there anything else you would like to tell us, or anything you would like to ask? Please feel free to contact us at any time if you have any questions.

Note: After the interview is completed prepare a note with information on how you arranged the SSI, the appearance of the house and environment (give an indication of their socioeconomic status), and any other information relevant. Ask for a permission to take a photograph of them or their home and village.

Semi-structured interview guide for health service providers

Date of interview: ……………………

Location of interview: ……………………

Interviews start time: ……………………

Key informant number: …………………….

Inclusion criteria: Health workers from Dhanusha district.

Consent: Prior to beginning the interview, participants will be read the consent form and give written consent and an oral consent on an audio recording. Give the participant a copy of the consent form and the information sheet.

Introduction: Interviewer should explain the study purpose to the participant and answer any questions he/she might have.

Collect some information about the participant:

- Participant name:
- Participant age:
- Participant gender:
- Participant caste:
- Highest level of education attained:
- Occupation:
- Years in the current position:
- Contact number:

Instructions:
Use these questions to guide your conversation with the participants and probe where necessary. Statements in ***bold*** *inform the participant about the topic of conversation. Indented questions are follow-up or probing questions.*

Availability and experiences of nutrition services

First, I would like to ask about the nutrition services currently provided to children under three.

a. What nutrition programmes are implemented by the government health services for children under three years of age?
b. Can you tell me about the nutrition programmes implemented for young children?
    i. What kind of services are provided?
    ii. What are the activities involved?
    iii. Who provides the services?
    iv. How are the financial resources managed?
c. What are the things that has made it easy for the delivery of nutrition programmes at the facility/community?
    i. What has made it difficult?
d. What are the things that has made it easy for community people to participate and follow the advice provided through nutrition programmes?

i. What has made it difficult?

Availability of stimulation (play and learning) services

Now I would like to ask about the play-related programmes for children under three years age at the health facility and community.

a. What kind of support is there to improve play and learning for children in the local area? (For e.g., informal playgroup in the community)
b. In your view, what kind of support is needed to improve play and learning-related practices?

Integration of playing (stimulation) and nutrition services

Now I would like to ask you about what happens if stimulation/playing is combined with the existing nutrition services

The World Health Organization (WHO) has announced a new guideline to improve child development. The WHO recommends nutrition and playing and learning interventions for children below three years of age to improve their growth and development.

a. What are your opinions about adding play and learning-related activities to the current nutrition programme (give example)?
b. What nutrition programmes/services are there that could provide an opportunity for adding play and learning-related activities?
c. How do you think the existing nutrition programme provides an opportunity for integration?
d. In your view, how would people in the community see the introduction of play and learning activities along with an ongoing nutrition programme?
e. What are the effects of the delivery of combined nutrition and playing/learning intervention on the young children in the community?

Intervention implementation

Now I would like to ask about who should be involved in the delivery of the combined nutrition and play and interaction intervention.

If play and learning-related services were to be added to nutrition services:

a. How should the combined nutrition and playing/learning-related activities be delivered to the community?
b. Who should participate? (Probe about the family members. What are the reasons? How would their participation affect child's learning and their childcare practices?)
c. What works are involved in the implementation?
    i. Who do you think should deliver the programme?
        1. What is the reason for choosing that health care provider?
        2. Who could be the alternative? (If not the existing health worker) Why?
        3. What kind of knowledge would be necessary?
        4. What kind of support will they need to perform the task? (Training, supervision, financial)
        5. What changes will the new task bring in their roles? (If current staff)
        6. How will this affect their workload?
        7. How will be the workload managed? (Probe addition of new category, how will they be managed?)
        8. What could be the alternatives? Why?
d. Which modality could be used to deliver the intervention effectively?
    i. What are the reasons?

e. Who should participate in the programme from the community to make it effective? Why?
f. What are the factors that would motivate the existing service providers to perform the new play and learning-related tasks in addition to the existing nutrition services?
    i. What are the barriers that would prevent service providers from performing the task?
g. Who do you think should be involved in making changes (addition of play and learning activities with current nutrition-related activities) happen?
    i. How can they support the programme?
    ii. What kind of support they could be provided?

Resources for support

Now I would like to ask about the resources available to support the integration.

If play and learning-related services were to be added to nutrition services:

a. What are the financial resources available?
    i. What could be the alternatives? Why?
b. How could financial resources affect the ability of the health system?

Challenges

Now I would like to ask about the challenges in the integration.

If play and learning-related services were to be added to nutrition services:

a. In your opinion, in the implementation of combined nutrition and stimulation programme (i.e., adding the play and learning-related activities in the current nutrition programme)
    i. What would make it easy?
    ii. What challenges do you think there could be?
b. What are the things that might make it easy for community people to participate and follow the advice provided in the combined programme? (Probe for social and cultural barriers)
    i. What might make it difficult?
c. How do you think community people could contribute to the implementation of the programme? What could be their role?
d. In the context of the current health system in Nepal, what strategies could contribute to the sustainability of such interventions?
e. In our interview with community people and health workers, they talked about the support and help of the NGO sector.
    - What are your views regarding this?
    - What could be the role of the NGO sector?
    - How will this affect sustainability?

Conclusion: Thank you very much for your time. Is there anything else you would like to tell us, or anything you would like to ask? Please feel free to contact us at any time if you have any questions.

Note: After the interview is completed prepare a note with information on how you arranged the SSI, the appearance of the environment (give an indication of their setup), and any other relevant information. Ask for permission to take a photograph of them or their settings.

Semi-structured interview guide for stakeholders and policymakers

Date of interview: ……………………

Location of interview: ……………………

Interviews start time: ……………………

Key informant number: …………………….

**Inclusion criteria:** National and district stakeholder representative from health and education sector.<br><br>**Consent:** Prior to beginning the interview, participants will be read the consent form and give written consent on an audio recording. Give the participant a copy of the consent form and the information sheet.<br><br>**Introduction:** Interviewer should explain the study purpose to the participant and answer any questions he/she might have.

Collect some information about the participant:

- Participant name:
- Participant age:
- Participant gender:
- Participant caste:
- Highest level of education attained:
- Occupation:
- Years in the current position:
- Contact number:

Instructions:
Use these questions to guide your conversation with the participants and probe where necessary. Statements in ***bold and italics*** *inform the participant about the topic of conversation. Indented questions are follow-up or probing questions.*

ECD policy in Nepal:

First, I would like to ask you about policies, rules and regulations regarding early childhood development.

1. Can you tell me about the Nepal government's policy, rules and regulations regarding early childhood development?
2. How much of the existing policy has been implemented?
    - What has been done to improve the stimulation practices of young children in Nepal? (Probe for children three years and above and below three years)
3. What are the barriers to the implementation of current ECD policies?
    - How can it be made easy?

Integration of nutrition and stimulation interventions:

Now I would like to ask you what happens if stimulation/playing is combined with the existing nutrition services.

The World Health Organization (WHO) has announced a new guideline to improve child development. The WHO recommends combined nutrition and stimulation interventions for children below three years.

1. What is your perception of the feasibility of the implementation of a combined intervention in Nepal?
    - What are the advantages and disadvantages?
2. What nutrition services for children under three years could provide an opportunity for integration?
    - How will it provide the opportunity?
3. What are the barriers/challenges to the integration of stimulation intervention in the nutrition programme in the health services? (For example, social and political constraints.)
4. What are the facilitators for the integration of stimulation intervention in the nutrition programme in health services?

Implementation of integrated intervention:

Now I would like to ask about the delivery of the combined nutrition and play, and interaction intervention.

If the integration were to happen:

1. We have a federal structure in Nepal, and the local governments has already been formed. In this case, how will the integrated intervention be managed and delivered?
2. What would be the role and responsibility of the health post in the implementation of integrated stimulation and nutrition intervention in the community?
    - What could be an issue related to the implementation of stimulation and nutrition-related services, i.e., service use, access, and quality?
    - How much sufficient are human resources to implement stimulation-related activities?
    - How can health workers be included in the delivery of such programmes?
    - What do you think about the potential adoption of this intervention into the usual care of community health workers?
    - What are the financial resources available for nutrition and early childhood development?
        - What could be the alternatives? Why?
        - How could financial resources affect the ability to integrate services into facility-based / community-based services?
3. In the context of the current health system in Nepal, what strategies could contribute to the sustainability of such interventions?
4. In our interview with community people and health workers, they talked about the support and help of the NGO sector.
    - What are your views regarding this?
    - What could be the role of the NGO sector?
    - How will this affect sustainability?

Thank you very much for your time. Is there anything else you would like to tell us or anything you would like to ask?

Focus group discussion guide for FCHVs and HFOMC members

Date of interview: ……………………

Location of interview: ……………………

Interviews start time: ……………………

Key informant number: …………………….

Inclusion criteria: Female community health volunteers and members of Health facility management committee in Dhanusha district.

Consent: Prior to beginning the interview, participants will be read the consent form and give written consent and an oral consent on an audio recording. Give the participant a copy of the consent form and the information sheet.

Introduction: Interviewer should explain the study purpose to the participant and answer any questions he/she might have.

Collect some information about the participant:

- Participant name:
- Participant age:
- Participant gender:
- Participant caste:
- Highest level of education attained:
- Occupation:
- Years in the current position:
- Contact number:

Instructions:
*Use these questions to guide your conversation with the participants and probe where necessary. Statements in **bold** inform the participant about the topic of conversation. Indented questions are follow-up or probing questions.*

Availability and experiences of nutrition services

First, I would like to ask about the nutrition services currently provided to children under three.

e. What nutrition programmes are implemented by the government health services for children under three years of age?
f. Can you tell me about the nutrition programmes implemented for young children?
    v. What kind of services are provided?
    vi. What are the activities involved?
    vii. Who provides the services?
    viii. How are the financial resources managed?
g. What are the things that has made it easy for the delivery of nutrition programmes at the facility/community?
    i. What has made it difficult?
h. What are the things that has made it easy for community people to participate and follow the advice provided through nutrition programmes?

    i. What has made it difficult?

Availability of stimulation (play and learning) services

Now I would like to ask about the play-related programmes for children under three years of age at the health facility and community.

c. What kind of support is there to improve play and learning for children in the local area? (E.g., informal playgroup in the community)
d. In your view, what kind of support is needed to improve play and learning-related practices?

Integration of playing (stimulation) and nutrition services

Now I would like to ask what happens if stimulation/playing is combined with the existing nutrition services.

The World Health Organization (WHO) has announced a new guideline to improve child development. The WHO recommends nutrition, playing, and learning interventions for children below three years of age to enhance their growth and development.

f. What are your opinions about adding play and learning-related activities to the current nutrition programme (give an example)?
g. What nutrition programmes/services could provide an opportunity for adding play and learning-related activities?
h. How does the existing nutrition programme provide an opportunity for integration?
i. In your view, how would people in the community see the introduction of play and learning activities along with an ongoing nutrition programme?
j. What are the effects of the delivery of combined nutrition and playing/learning intervention on the young children in the community?

Intervention implementation

Now I would like to ask who should be involved in the delivery of the combined nutrition and play and interaction intervention.

If play and learning-related services were to be added to nutrition services:

h. How should the combined nutrition and playing/learning-related activities be delivered to the community?
i. Who should participate? (Probe about the family members. What are the reasons? How would their participation affect child's learning and their childcare practices?)
j. What works are involved in the implementation?
    i. Who do you think should deliver the programme?
        1. What is the reason for choosing that health care provider?
        2. Who could be the alternative? (If not the existing health worker) Why?
        3. What kind of knowledge would be necessary?
        4. What kind of support will they need to perform the task? (Training, supervision, financial)
        5. What changes will the new task bring in their roles? (If current staff)
        6. How will this affect their workload?
        7. How will the workload be managed? (Probe addition of new category, how will they be addressed?)
        8. What could be the alternatives? Why?
k. Which modality could be used to deliver the intervention effectively?
    i. What are the reasons?
l. Who should participate in the programme from the community to make it effective? Why?

m. What factors would motivate the existing service providers to perform the new play and learning-related tasks in addition to the current nutrition services?
    i. What are the barriers that would prevent service providers from performing the task?

n. Who do you think should be involved in making changes (addition of play and learning activities with current nutrition-related activities) happen?
    i. How can they support the programme?
    ii. What kind of support could they be provided?

Resources for support

Now I would like to ask about the resources available to support the integration.

If play and learning-related services were to be added to nutrition services:

c. What are the financial resources available?
    i. What could be the alternatives? Why?

d. How could financial resources affect the ability of the health system?

Challenges

Now I would like to ask about the challenges in the integration.

If play and learning-related services were to be added to nutrition services:

f. In your opinion, in the implementation of combined nutrition and stimulation programme (i.e., adding the play and learning-related activities in the current nutrition programme)
    i. What would make it easy?
    ii. What challenges do you think there could be?

g. What are the things that might make it easy for community people to participate and follow the advice provided in the combined programme? (Probe for social and cultural barriers)
    i. What might make it difficult?

h. How could community people contribute to the implementation of the programme? What could be their role?

i. In the context of the current health system in Nepal, what strategies could contribute to the sustainability of such interventions?

j. In our interview with community people and health workers, they talked about the support and help of the NGO sector.
    - What are your views regarding this?
    - What could be the role of the NGO sector?
    - How will this affect sustainability?

Conclusion: Thank you very much for your time. Is there anything else you would like to tell us, or anything you would like to ask? Please feel free to contact us at any time if you have any questions.

Note: After the discussion is completed, prepare a note with information on how you arranged the discussion, the appearance of the environment (give an indication of the setup), and any other relevant information. Ask for permission to take a photograph of them or their settings.
