# Supplementary material for: Exploring the feasibility of integrating health, nutrition and stimulation interventions for children under three years in Nepal’s health system: A qualitative study
Source: PLOS Glob Public Health. 2023 Apr 28;3(4):e0001398. doi: 10.1371/journal.pgph.0001398 (PMC10146516; doi:10.1371/journal.pgph.0001398)
Supplement: S2 File — (PDF) [file pgph.0001398.s004.pdf]

S2 File: Vignette

Children learn through play

Objectives:

- To discuss the activities that children do in the first three years of life and what they learn from them.
- To introduce the concept of stimulation using a game.
- To identify the local term used by participants to describe stimulation.

Materials (locally available low-cost toys):

1. A bottle with a screw lid
2. Two bangles
3. Two pieces of square cloth
4. A cup and a small plate
5. A shop-bought toy or any item easily identifiable as a toy- doll, ball, rattle

Activity 1. Brainstorming:

Ask participants to bring their children/grandchildren (three or less than three years old) and play materials they have at home that their children can use as play materials. Then ask them to think about and discuss the following:

1. What kinds of games do children play in each of the materials or things in the first three years of life?
2. For each game mentioned, ask, “What do children learn when they play like this?” Prompt ‘What skills – physical, mental, language, and socio-emotional?’

If participants do not know/talk about any types of games that children can play with toys, use the demonstration method (show different types of play activities using materials gathered by the participant or provide your own toys) and ask:

1. For each game demonstrated, ask, “What do children learn when they play like this?” Prompt ‘What skills – physical, mental, language, and socio-emotional?’

Activity 2. Playing games:

Play a game to help the participant understand 1) how young children learn through play and 2) the importance of play in children’s growth and development.

Instruction of game: Ask a participant to sit comfortably with their child. Please give them a task to play games with their child using their toys. If the child does not have any toys, provide them with locally available age-appropriate toys such as bangles, bottles with caps, two pieces of cloth, or a ball. Facilitate and encourage participants if they do not do any activities with their children.

Discuss:

Ask participants:

a. How did you feel playing with your child?
b. What was your child’s response to this play activity?
c. Can you give me examples of similar games you play at home with your child (shown in Activity 2)?
d. Can you provide examples of games you play with your child without using toys at home?
e. To what extent have you and other household members been doing such play activities as we did with your child?
f. What do you think children learn from playing these games (with or without using toys)?
g. What might be the effect on a child that does not have the chance to play games of this nature?

Summarise the game:

When the participant (mother/father/grandmother) and child played together using toys, they enjoyed and learned new things. Besides, they got additional support and ideas. Similarly, younger children who get the opportunity to be involved in different play activities enjoy and learn new things more quickly than younger children who don’t get the opportunity to involve in other play activities. The learning process becomes slow in children who do not involve in such play activities. Therefore, play activities make the learning process quicker in younger children and help in their growth and development.
