# Supplementary material for: Exploring the feasibility of integrating health, nutrition and stimulation interventions for children under three years in Nepal’s health system: A qualitative study
Source: PLOS Glob Public Health. 2023 Apr 28;3(4):e0001398. doi: 10.1371/journal.pgph.0001398 (PMC10146516; doi:10.1371/journal.pgph.0001398)
Supplement: S3 File — Developed from: Tong A, Sainsbury P, Craig J. Consolidated criteria for reporting qualitative research (COREQ): A 32-item checklist for interviews and focus groups. International Journal for Quality in Health Care. 2007. Volume 19, Number 6: pp. 349–357. (PDF) [file pgph.0001398.s005.pdf]

S3 File: Consolidated Criteria for Reporting Qualitative Research (COREQ) checklist

Exploring the feasibility of integrating health, nutrition, and stimulation interventions for children under three years in Nepal's health system: a qualitative study

**Domain 1: Research team and reflexibility**	
Personal Characteristics:<br>1. Interviewer/facilitator,<br>2. Credential<br>3. Occupation<br>4. Gender<br>5. Experience and training	Researcher KG conducted all individual interviews and focus group discussions with caregivers and health care providers in Maithili, except for two interviews, which were conducted in Nepali. Likewise, SD conducted all individual interviews with stakeholders and policymakers in Nepali except one in English.<br><br>AP is a Professor of Global Health at University College London, Institute for Global Health in London. NSM is a Principal Research Associate at University College London, Institute for Global Health, primarily based in Nepal. DM is a Professor of Public Health at the School of Health Sciences, Western Sydney University in Australia. SD is a PhD candidate at Western Sydney University in Australia. KG has completed higher secondary education and worked as a qualitative researcher in Nepal.<br><br>All research teams involved in this study are females.<br><br>All research team members have extensive experience and training in conducting qualitative research.
Relationship with participants<br>6. Relationship established<br>7. Participant knowledge of interviewer<br>8. Interviewer characteristics	SD and KG did not know the participants prior to the data collection. They do not have any affiliation or relationship with the participants.<br><br>Participants were introduced to the researcher before the data collection. They were informed about the purpose of the researcher's visit to their homes or workplace and described the study.<br><br>SD and KG are Nepalese researchers.<br><br>SD undertook this research as a part of her PhD. SD is fluent in Nepali and English and has a basic understanding of spoken Maithili but required transcripts to be translated into Nepali and English for analysis. SD lived and worked in Dhanusha between 2012 and 2016. SD has over 10 years of experience in qualitative and quantitative research on child health, nutrition, and development.<br><br>KG is from Dhanusha, has completed higher secondary education, is fluent in Nepali and Maithili and has over 15 years of research experience.<br><br>SD and KG were aware that while interviewing participants, they had to remain neutral and listen from a researcher's perspective.

**Domain 2: Study design**	
Theoretical framework<br>9. Methodological orientation and Theory	The World Health Organisation (WHO) health system framework and Nonadopting, Abandonment, Scale-up, Spread, and Sustainability (NASSS) framework were used to guide the development of the topic guide.<br><br>Framework approach was used for data analysis.
Participant selection<br>10. Sampling,<br>11. Method of approach<br>12. Sample size<br>13. Non-participation	A purposive sampling approach was used to recruit participants.<br><br>We identified caregivers after consulting a local Female Community Health Volunteer, who introduced us to participants and their family members. We consulted with municipality leaders, and health post in charge, to identify health service providers. We identified national and district stakeholders and policymakers after consulting governmental and non-governmental organisation employees.<br><br>We conduced 30 individual interviews and four Focus Group Discussions (FGDs).<br><br>Two participants (a father and mother) refused to participate in the interview because they felt shy to communicate with us.
Setting<br>14. Setting of data collection,<br>15. Presence of non-participants,<br>16. Description of sample	Caregivers were interviewed in their homes and health service providers and district stakeholders at their workplaces. All interviews with stakeholders were conducted online via Zoom, except for three, which were conducted in person at participants' workplaces.<br><br>All interviews were conducted privately.<br><br>Sample characteristics of participants from interview and focus group discussions are reported in results section in Table 1 and 2
Data collection<br>17. Interview guide,<br>18. Repeat interviews,<br>19. Audio/visual recording,<br>20. Field notes,<br>21. Duration,<br>22. Data saturation,<br>23. Transcripts returned	The WHO health system framework and NASSS framework were used to guide the development of the topic guide. Topic guides were pretested and refined iteratively. All topic guides were developed in Nepali and Maithili.<br><br>No repeat interviews were conducted.<br><br>All interviews were audio recorded and stored on a password protected device. Informed written consents were obtained prior to audio recordings.<br><br>SD wrote field notes during interviews and discussions.<br>Interviews averaged 75 (47-117) minutes, and focus group discussions averaged 133 (127-140) minutes in length.

	We stopped data collection when data saturation was achieved.<br><br>No transcripts were returned to participants.
**Domain 3: Analysis and findings**	
Data analysis<br>24. Number of data coders<br>25. Description of the coding tree<br>26. Derivation of themes<br>27. Software<br>28. Participant checking	AP, DM and SD coded selected three thematically representative transcripts separately first to developed preliminary codes. AP, DM and SD coded these separately and met to discuss and agree on preliminary codes. We deductively and inductively developed themes and developed an analytic framework. SD then used this framework to index all the transcripts.<br><br>Themes were defined from the interview guide, literature and emergence of themes from the participants.<br><br>SD index all the transcripts in NVIVO v20 software.<br><br>We did not conduct participants checking.
Reporting<br>29. Quotations presented<br>30. Data and findings consistent<br>31. Clarity of major themes,<br>32. Clarity of minor themes	We report the results using illustrative quotes for themes identified in the results. We have also provided additional quotations in supplementary file 5. Participants were kept anonymous. We numbered each interview and FGD by number, for example, Father SSI 1.<br><br>We checked for consistency between the data and findings iteratively by comparing transcripts, summary chart and audio-recording where necessary.<br><br>Major and minor themes are clearly identified and discussed in the result section.

Developed from: Tong A, Sainsbury P, Craig J. Consolidated criteria for reporting qualitative research (COREQ): a 32-item checklist for interviews and focus groups. International Journal for Quality in Health Care. 2007. Volume 19, Number 6: pp. 349 – 357
