# Supplementary material for: Exploring the feasibility of integrating health, nutrition and stimulation interventions for children under three years in Nepal’s health system: A qualitative study
Source: PLOS Glob Public Health. 2023 Apr 28;3(4):e0001398. doi: 10.1371/journal.pgph.0001398 (PMC10146516; doi:10.1371/journal.pgph.0001398)
Supplement: Attachment — Submitted filename: Response to Reviewers.docx [file pgph.0001398.s006.docx]

**Response to the Reviewers**

**Journal Requirements:**

**1. Please review your reference list to ensure that it is complete and correct. If you have cited papers that have been retracted, please include the rationale for doing so in the manuscript text or remove these references and replace them with relevant current references. Any changes to the reference list should be mentioned in the rebuttal letter that accompanies your revised manuscript. If you need to cite a retracted article, indicate the article’s retracted status in the References list and also include a citation and full reference for the retraction notice.**

**Author’s response:** We apologise for the mistakes in the reference list. We have checked the reference list and revised it according to the journal’s guidelines. We have mentioned the changes below:

1. We have removed additional information such as Epub, PubMed PMID, PubMed Central PMCID wherever listed in the references
2. The numbering and order of references has been changed after the revisions.
3. We have added the following information to references that were incomplete:

Added page number:

7. Dulal S, Prost A, Karki S, Saville N, Merom D. Characteristics and effects of integrated nutrition and stimulation interventions to improve the nutritional status and development of children under 5 years of age: a systematic review and meta-analysis. BMJ global health. 2021;6:e003872. doi: 10.1136/bmjgh-2020-003872.

Added volume and page number:

26. Goldsmith L. Using Framework Analysis in Applied Qualitative Research. The Qualitative Report. 2021;26:2061-76. doi: 10.46743/2160-3715/2021.5011.

49. Shrestha N, Mishra SR, Ghimire S, Gyawali B, Marahatta SB, Maskey S, et al. Health System Preparedness for COVID-19 and Its Impacts on Frontline Health-Care Workers in Nepal: A Qualitative Study Among Frontline Health-Care Workers and Policy-Makers. Disaster Medicine and Public Health Preparedness. 2021;16:2560-8. doi: 10.1017/dmp.2021.204.

Added online link and accessed date:

29. Walker SP, Powell P, Chang SM, Baker-Henningham H, Grantham-McGregor SM, Vera-Hernandez M, et al. Delivering Parenting Interventions Through Health Services in the Caribbean.2019 [cited 2022 October 15]. Available from: <https://docs.iza.org/dp12106.pdf>.

32. Woodhead M, Feathersone I, Bolton L, Robertson P. Early Childhood Development: Delivering Inter‐sectoral Policies, Programmes and Services in Low‐resource Settings. Topic guide [Internet]. 2014 [cited 2022 October 10]. Available from: <http://oro.open.ac.uk/41552/1/Woodhead%20et%20al%202014%20Early-Childhood-Development-Topic-Guide.pdf>.

48. Kitsao-Wekulo P, Haycraft E, Mwoma T, Okelo K, Kinuthia E, Muriuki P, et al. Cultural and contextual considerations in designing programs to support the incorporation of early childhood development within ongoing national nutrition programmes: A focus on the Baby-Friendly Community Initiative (BFCI) in a rural African context. Research square [Internet]. 2021 30 November 2021 [cited 2022 October 10]. Available from: <https://assets.researchsquare.com/files/rs-1125126/v1/41d8d82d-1e2a-43c6-8497-ff964ac06104.pdf?c=1638298266>.

Corrected the editors name and added the chapter page number:

63. Fernald LC, Gertler PJ, Hidrobo M. Conditional cash transfer programs: Effects on growth, health, and development in young children. In: King R, Maholmes V, editors. The Oxford handbook of poverty and child development. Oxford: Oxford University Press; 2012. p. 569-600.

Added one new reference:

30. Shah R, Herekar V, Metgud D, Kim H, Atkins M, Dhaded S. Implementing an early childhood development intervention with routine immunization visits in India: a feasibility trial. Eur J Pediatr. 2022;181(7):2799-808. doi: 10.1007/s00431-022-04485-w.

Removed three references that were not cited in the manuscript. We apologise for the mistake.

Strachan D, Kabwijamu L, Okuga M, Daniel-Howell C, Haghparast-Bidgoli H, Nantanda R, et al. Shifting the discourse from survive to thrive: a qualitative exploration of beliefs, actions and priorities for early childhood development in Uganda. Journal of the British Academy. 2020;8s2:41-70. doi: 10.5871/jba/008s2.041.

Hollowell J, Dumbaugh M, Belem M, Kousse S, Swigart T, Korsaga C, et al. 'Grandmother, aren't you going to sing for us?' Current childcare practices and caregivers' perceptions of and receptivity to early childhood development activities in rural Burkina Faso. BMJ Glob Health. 2019;4(2):e001233. Epub 2019/04/19. doi: 10.1136/bmjgh-2018-001233. PubMed PMID: 30997165; PubMed Central PMCID: PMCPMC6441292.

Gladstone M, Phuka J, Mirdamadi S, Chidzalo K, Chitimbe F, Koenraads M, et al. The care, stimulation and nutrition of children from 0-2 in Malawi—Perspectives from caregivers; "Who’s holding the baby?". PLOS ONE. 2018;13(6):e0199757.

**2. In the online submission form, you indicated that "The fully anonymised data will be shared on reasonable request with the corresponding author". All PLOS journals now require all data underlying the findings described in their manuscript to be freely available to other researchers, either: 1. In a public repository, 2. Within the manuscript itself, or 3. Uploaded as supplementary information.**

This policy applies to all data except where public deposition would breach compliance with the protocol approved by your research ethics board. If your data cannot be made publicly available for ethical or legal reasons (e.g., public availability would compromise patient privacy), please explain your reasons by return email and your exemption request will be escalated to the editor for approval. Your exemption request will be handled independently and will not hold up the peer review process but will need to be resolved should your manuscript be accepted for publication. One of the Editorial team will then be in touch if there are any issues.

**Author’s response:** We have now published anonymised data (full transcripts in English) in the repository of Western Sydney University. We have added the link on the data availability and will also provide this information online while re-submitting the revisions. We have also provided the data related to the findings in the S2 Table.

“Data supporting the findings are available in the article’s supplementary material (S2 Table). The fully anonymised data can be accessed online in the Western Sydney University repository via the link: <https://doi.org/10.26183/j49n-0g80>”

[Note: HTML markup is below. Please do not edit.]

**Reviewers’ comments:

Reviewer’s Responses to Questions**

**Comments to the Author**

1. Does this manuscript meet PLOS Global Public Health’s [publication criteria](https://journals.plos.org/globalpublichealth/s/criteria-for-publication)? Is the manuscript technically sound, and do the data support the conclusions? The manuscript must describe methodologically and ethically rigorous research with conclusions that are appropriately drawn based on the data presented.

Reviewer #1: Yes

Reviewer #2: Yes

2. Has the statistical analysis been performed appropriately and rigorously?

Reviewer #1: N/A

Reviewer #2: N/A

3. Have the authors made all data underlying the findings in their manuscript fully available (please refer to the Data Availability Statement at the start of the manuscript PDF file)?

The [PLOS Data policy](https://journals.plos.org/globalpublichealth/s/data-availability) requires authors to make all data underlying the findings described in their manuscript fully available without restriction, with rare exceptions. The data should be provided as part of the manuscript or its supporting information or deposited to a public repository. For example, in addition to summary statistics, the data points behind means, medians and variance measures should be available. If there are restrictions on publicly sharing data—e.g., participant privacy or use of data from a third party—those must be specified.

Reviewer #1: No

Reviewer #2: Yes

4. Is the manuscript presented in an intelligible fashion and written in standard English?

PLOS Global Public Health does not copyedit accepted manuscripts, so the language in submitted articles must be clear, correct, and unambiguous. Any typographical or grammatical errors should be corrected at revision, so please note any specific errors here.

Reviewer #1: Yes

Reviewer #2: Yes

**5. Review Comments to the Author**

**Author’s note:** The changes in the manuscript as shown in the responses below are underlined.

**Reviewer #1**

Overall, this is an excellent paper. It tackles an important topic and is well designed, and based on evidence of a strong report, very well executed overall. It would make a valuable contribution to the literature and would be a good fit for PLOS GH. My comments are thus very minor:

**Author’s response:** Thank you to the reviewer for the positive feedback.

**A. Sampling**

1. **Can you say a little more about how the participants were identified. Were the local FCHVs known to you; did they know the families and what was the chance they picked families whom they thought might be pro-ECD?**

**Author’s response:** We thank you for the comment. We have added the following details under the sub-heading Sampling on page number 7:

“Local FCHVs helped us to identify participants based on the inclusion criteria and introduced us to participants and their family members. SD did not know any FCHVs from the study site. KG was familiar with three FCHVs from the urban municipality. We did not disclose the topic for the interview with FCHVs prior to the participant identification.”

2. **Likewise, were the policymakers known to the research team or was this the first interaction?**

**Author’s response:**  We have added a sentence describing that the interviewers had no relationship with the participants under the sub-heading Reflexivity on page number 11:

“SD and KG did not know the participants and do not have any affiliation or relationship with them prior to the data collection.”

3. **Please say a little more about SD and KG who (if I understood right) did the interviews. For example, prior experience; background (see COREQ re ‘reflexivity of the research team)**

**Author’s response:** Thank you for this suggestion. We have added a section on reflexivity where we describe the interviewer’s credentials, occupation, experience and training under the sub-heading Reflexivity on page number 11.

“This research form’s part of SD’s PhD. SD is a Nepalese female researcher with a nursing background, fluent in Nepali and English and with over ten years of research experience, including three years living in Dhanusha. SD has a basic understanding of spoken Maithili but required transcripts to be translated into Nepali and English for analysis. KG has completed higher secondary education and is a trained female qualitative researcher from Dhanusha. She is fluent in Nepali and Maithili, with over 15 years of research experience. SD and KG did not know the participants and do not have any affiliation or relationship with them prior to the data collection. They maintained neutrality and kept their personal opinions and reactions aside during data collection and analysis.”

4. **Did KG interpret all for SD or did SD also speak some Nepali? Was there discussion between SD and KG during interviews so as to steer next questions based on previous ones or did KG do all with SD just observing.**

**Author’s response:** We have clarified about the number of interviews conducted by KG and SD, the supervision of interviews/FGDs done by KG under the sub-heading Data collection on page number 9.

“KG conducted all SSIs with caregivers (n=17) and health service providers (n=3) and FGDs with FCHVs (n=2) and HFOMC members (n=2) in the local language (Maithili), except for two SSIs (with a father and health coordinator) which were conducted in Nepali. SD conducted all SSIs with national (n=3) and district (n=1) stakeholders and policymakers (n=3) in Nepali, except for one (with national stakeholder) in English. The language was based on the participant’s preference.

…

SD observed and supervised KG during data collection and provided feedback after each SSI or FGD. SD did not suggest probes or follow-up questions during interviews as KG was well trained in formulating these”.

and about the credentials of SD the sub-heading Reflexivity on page number 11.

“This research form’s part of SD’s PhD. SD is a Nepalese female researcher with a nursing background, fluent in Nepali and English and with over ten years of research experience, including three years living in Dhanusha. SD has a basic understanding of spoken Maithili but required transcripts to be translated into Nepali and English for analysis.”

5. **Figure 1 is good – though the resolution is poor so I couldn’t see all details. Not essential but consider making this more visually appealing with perhaps some further subheadings to summarise key points.**

**Author’s response:** Thank you for your positive feedback. I have revised the resolution of Figure 1 as suggested by the journal, file format TIFF, width is 2250 pixels and resolution is 600 dpi pixels/inch. We did not add further subheadings to the key points because we fear the figure will look crowded as it is already rich in text. Since the barriers and facilitators are discussed within each theme in the text and not separately, the idea was to show them collectively in a figure at three levels of influence and then list them as explicitly as possible.

We have also added Table 3 to provide a summary of the themes and sub-themes identified so that the reader can cross-refer between figure 1 and Table 3 for more detail.

**B. Discussion**

1. **Please say more about the potential role of ECD integrated in different TYPES of health programme.**

For instance, I can see a big opportunity when infants and children come for immunization visits or growth monitoring visits and may otherwise just be waiting around for quite a while before a short interaction with a HCW Likewise, there are long periods of inactivity during a CMAM nutrition clinic which could be well utilised by ECD activities and education. However, linking with IMCI may be more problematic and maybe not even desirable, at least in same way since by definition the children are unwell, clinical priorities should take 1st place and if sick, both children and parents are less likely to engage with ECD.

**Author’s response:** We thank you for this comment. We have now explained this under Discussion on page number 31.

**“**Healthcare visits that often involve long waiting times, for instance, immunisation or monthly growth monitoring programmes, are better suited to offer ECD activities [29, 30] than rapid ones with limited opportunities for interaction with healthcare workers.

**Reviewer #2:** The article aims to understand the feasibility of integrating an ECD intervention for children below 3 years of age to the health system in Nepal. With the study the authors try to understand the barriers and enablers to fill an existing gap which is the delivery of ECD interventions for young children in Nepal. The manuscript has some important merits: A robust qualitative methodology that includes data collection from beneficiaries, providers, managers, and policymakers. Even though the findings have mainly a local interest, the authors raise an important aspect of the implementation barriers regarding cultural aspects that are common to other countries. The manuscript is also very well written; however, it is very long. I suggest revising and trying to shorten, especially the results section.

**Author response:** Thank you for your positive feedback. We have revised and shortened the results section. The word count has changed slightly from 7401 to 7246. However, due to new additions made during revision, the word count remains similar.

**There are minor issues that also should be addressed:**

1. **There is very limited information about the interviewers’ credentials, occupation, experience, and training. The authors didn’t include a report the interviewer’s relationship with the participants if existent.**

**Author’s response:** Thank you for highlighting this. As explained in our rebuttal to reviewer 1, we now describe the interviewer’s credentials, occupation, experience and training and the interviewer’s relationship with the participants under the sub-heading Reflexivity on page number 11.

“This research form’s part of SD’s PhD. SD is a Nepalese female researcher with a nursing background, fluent in Nepali and English and with over ten years of research experience, including three years living in Dhanusha. SD has a basic understanding of spoken Maithili but required transcripts to be translated into Nepali and English for analysis. KG has completed higher secondary education and is a trained female qualitative researcher from Dhanusha. She is fluent in Nepali and Maithili, with over 15 years of research experience. SD and KG did not know the participants and do not have any affiliation or relationship with them prior to the data collection. They maintained neutrality and kept their personal opinions and reactions aside during data collection and analysis.”

2. **The sample size selection is briefly described, and the authors did not mention participants’ dropouts or refusals.**

**Author’s response:** Thank you for your comment. We have added more detail including number of participants sampled from each category under the sub-heading Sampling on page number 7:

“From separate households, we sampled 18 caregivers (seven mothers, six fathers and five grandmothers) whose children/grandchildren were below three years for interviews. We purposively sampled caregivers from marginalised (Muslim and Dalit) and better-off caste groups and from poor and better-off households to explore a wide range of responses …

We consulted with municipality leaders and persons in charge of health posts to identify 18 health service providers: two health coordinators, one health assistant, and one Auxiliary Nurse Midwife (ANM) were sampled for interviews and seven FCHVs from each municipality were sampled for FGDs. Likewise, we identified representatives involved in the implementation of health, nutrition, and education-related programmes including four national and one district stakeholders after consulting governmental and non-governmental organisation employees and sampled them for interviews. We sampled Health Facility Operation and Management Committees (HFOMC) members, six from each municipality for FGDs. We also sampled three policymakers from the health and education sectors of the national government and senior provincial health service representatives for interviews.”

We have mentioned the participant’s refusal to participate in the study under the sub-heading Sampling on page number 8:

“Two caregivers (a father and mother) refused to take part in the interview because they felt shy to communicate with us.”
